# Prognostic Implication of M2 Macrophages Are Determined by the Proportional Balance of Tumor Associated Macrophages and Tumor Infiltrating Lymphocytes in Microsatellite-Unstable Gastric Carcinoma

**DOI:** 10.1371/journal.pone.0144192

**Published:** 2015-12-29

**Authors:** Kyung-Ju Kim, Xian-Yu Wen, Han Kwang Yang, Woo Ho Kim, Gyeong Hoon Kang

**Affiliations:** 1 Laboratory of Epigenetics, Cancer Research Institute, Seoul National University College of Medicine, Seoul, Korea; 2 Department of Pathology, Soonchunhyang University Cheonan Hospital, Cheonan, Korea; 3 Department of General Surgery, Seoul National University College of Medicine, Seoul, Korea; 4 Department of Pathology, and Seoul National University College of Medicine, Seoul, Korea; University of Nebraska Medical Center, UNITED STATES

## Abstract

Tumor associated macrophages are major inflammatory cells that play an important role in the tumor microenvironment. In this study, we investigated the prognostic significance of tumor associated macrophages (TAMs) in MSI-high gastric cancers using immunohistochemistry. CD68 and CD163 were used as markers for total infiltrating macrophages and M2-polarized macrophages, respectively. The density of CD68+ or CD163+ TAMs in four different areas (epithelial and stromal compartments of both the tumor center and invasive front) were analyzed in 143 cases of MSI-high advanced gastric cancers using a computerized image analysis system. Gastric cancers were scored as “0” or “1” in each area when the density of CD68+ and CD163+ TAMs was below or above the median value. Low density of CD68+ or CD163+ macrophages in four combined areas was closely associated with more frequent low-grade histology and the intestinal type tumor of the Lauren classification. In survival analysis, the low density of CD163+ TAMs was significantly associated with poor disease-free survival. In multivariate survival analysis, CD163+ TAMs in four combined areas, stromal and epithelial compartments of both tumor center and invasive front were independent prognostic indicator in MSI-high gastric cancers. In addition, the density of CD163+ TAMs correlated with tumor infiltrating lymphocytes (TILs). Our results indicate that the high density of CD163+ TAMs is an independent prognostic marker heralding prolonged disease-free survival and that the prognostic implication of CD163+ TAMs might be determined by the proportional balance of TAMs and TILs in MSI-high gastric cancers.

## Introduction

Gastric cancer (GC) is estimated to be the fifth most common malignancy in the world and is the third most common cause of death in both male and female individuals [1]. GC is a heterogeneous disease in terms of mechanisms of molecular carcinogenesis, and microsatellite instability (MSI) accounts for 10% of GCs [2, 3]. MSI is caused by genetic or epigenetic alterations of mismatch repair (MMR) genes and consequent alterations in the number of repeat nucleotides in coding or non-coding regions of the targeted genes. Compared with the MSI-low (MSI-L) or MS-stable (MSS) phenotype, MSI-high (MSI-H) GCs are characterized by some distinct clinicopathologic features, including more common GCs of intestinal type according to the Lauren classification, less frequent lymph node metastasis, and better prognosis [3]. At the same time, as a consequence of DNA mismatch repair deficiency, MSI-H GCs express many immunogenic antigens which lead to a high density of tumor infiltrating cytotoxic or regulatory T cells in the tumor stroma or tumor cells themselves [4, 5]. Consistent with the finding of tumor infiltrating lymphocytes (TILs), significantly enhanced numbers of tumor-associated macrophages (TAMs) have also been observed in MSI-H tumors compared with the MSS or MSI-L phenotype [6].

The tumor microenvironment plays a crucial role in many malignant tumors and involves several factors, including immune cells, fibroblasts, blood vessels, extracellular matrix, and soluble factors. Among them, macrophages are thought to be the most abundant immune populations. The major functions and characteristics of TAMs have been previously studied by many researchers. In general, TAMs release numerous factors such as cytokines, chemokines and growth factors that influence the behaviors of tumor cells. Monocytes are considered to have functional and phenotypic plasticity that enables them to differentiate into two polarization states—M1 and M2 macrophages—depending on the cytokine milieu in the tumor microenvironment [7]. Classically activated (M1) macrophages are induced by T helper type 1-like cytokines such as interferon-γ and microbial stimuli such as lipopolysaccharides and produce pro-inflammatory cytokines, chemokines and reactive nitrogen/oxygen intermediates. Thus, these cells are involved in anti-microbial and tumoricidal activity. In contrast, alternatively activated (M2) macrophages are induced by T helper type 2 cytokines including interleukin-4 (IL-4), IL-10 and IL-13 and show immunoregulatory, anti-inflammatory and tumor-promoting activity. In general, TAMs are considered to resemble the M2 phenotype more than the M1 phenotype [8]. Therefore, TAMs are thought to be associated with poor survival of cancer patients by promoting invasion, metastasis, angiogenesis and lymphangiogenesis. In fact, TAMs have been related to decreased survival in many solid tumors (e.g., ovary [9], melanoma [10], lung [11, 12], endometrium [13], breast [14, 15] and kidney [16, 17]) but not all (e.g., GCs, colorectal cancers (CRCs)). Several studies in GCs and CRCs have demonstrated better prognosis in patients with a high density of TAMs [18–21], which indicates that the functional role of TAMs could be different depending on type of tissue and cancer.

Because the molecular subtypes of GCs differ with regard to their clinicopathological features, including prognosis, in order to clarify the role of TAMs on the survival outcome in GCs, it is important to minimize the effect of confounding factors associated with prognosis and increase the study group’s homogeneity. MSI-H GCs are thought to provide an adequate platform to test whether TAMs are associated with good or poor survival. Thus, in the present study, a series of patients with MSI-H GCs was analyzed with regard to the number of infiltrated macrophages in the epithelial (E) and stromal (S) compartments of both the tumor center (TC) and invasive front (IF) regions with the use of tissue microarray-based immunohistochemistry and an image analyzer. We found that the high density of CD163-positive (CD163+) M2 macrophages in four combined areas, S and E compartment and IF region was invariably associated with prolonged disease-free survival (DFS) time in patients with MSI-H GCs.

## Material and Methods

The study protocol was reviewed and approved by the institutional review board of Seoul National University Hospital. Informed consent was exempted because of the retrospective nature of the study and minimal risk of harm to the study subjects. This study was performed in accordance with the recommendations of the Declaration of Helsinki for biomedical research involving human subjects. Patient records/information was anonymized and de-identified prior to analysis.

### Patients and specimens

We collected tissues from patients with GCs who received radical surgical resection with extended lymph node dissection at the Seoul National University Hospital, Seoul, Korea between 2004 and 2009. All samples were formalin-fixed paraffin-embedded (FFPE) tissues that had been submitted for pathological diagnosis. In our institution, MSI status was routinely analyzed in resected GC specimens by the molecular pathology laboratory. Among 1,706 cases of advanced GCs (AGCs), 160 cases (9.4%) were MSI-H GCs. In this study, AGC was defined by only its T stage which invades at least proper muscle layer (pT2 or more) regardless of the presence of lymph node metastasis referencing the previous studies [22, 23]. We excluded patients who at the time of surgery, had other malignancies or other critical medical problems. Finally, 143 cases were selected for this study. Clinicopathological information, including age, gender, tumor site, tumor differentiation, Lauren classification, Ming classification, TNM stage, body mass index (BMI), date of surgery, date of last follow-up and date of recurrence or death, were collected retrospectively from the electronic medical records. Among 143 patients, two patients could be classified as Lynch syndrome, previously termed Hereditary Non-Polyposis Colorectal Cancer (HNPCC), according to Amsterdam criteria-II. Follow-up periods (from surgery to death or the last follow-up) ranged from 2 to 111 months (median interval 70 months). Because cancer-related death rarely occurred owing to the good prognostic features of MSI-H GCs, we only evaluated DFS, which was defined as the duration in months from the date of surgery to death, tumor recurrence or date of the last follow-up. In this study, death or recurrence occurred in 30 cases (21%) out of all patients with MSI-H advanced GCs. Histologic grading and tumor staging were based on the American Joint Committee on Cancer (AJCC) Staging Manual Seventh Edition. This study was approved by the institutional review board of Seoul National University Hospital.

### DNA extraction and determination of MSI

The methods used for the MSI analysis have been previously described [24]. Briefly, manually micro-dissected tumor samples were digested with lysis buffer (100 mM Tris-HCl, 0.5% Tween-20, 1 mM EDTA and 20 μg/ml proteinase K) and incubated for 24 to 48 hr at 55°C until the tissue-containing lysis buffer cleared. Then, samples were incubated at 95°C for 10 min to inactivate proteinase K. Extracted genomic DNA was stored at -20°C until further use. MSI status was analyzed using markers according to the National Institutes of Health guidelines (BAT25, BAT26, D2S123, D5S346 and D17S250). We classified tumors as MSI-H when two or more markers showed instability, MSI-L when one marker showed instability, and MSS when none of the markers were unstable.

### GC tissue Microarray and Immunohistochemistry

**Fig 1:**
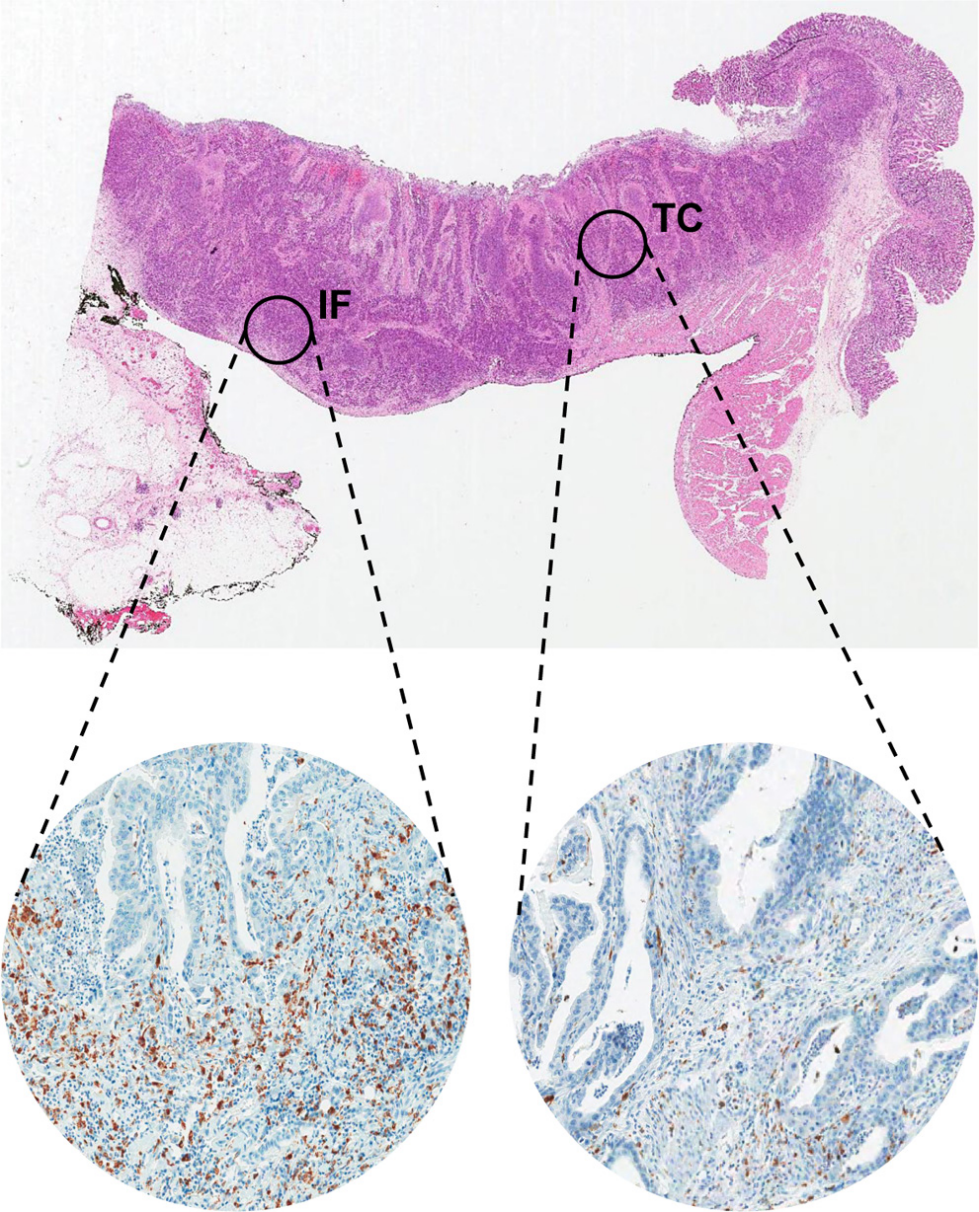
Representative image of the IF and TC in MSI-H advanced GCs. H&E section of GCs (original magnification, 12.5x) (top) showing each regions of the tumor: IF and TC. Immunohistochemical staining for CD68 and CD163 in each region (bottom). *Abbreviations*: IF, invasive front; TC, tumor center; MSI-H, microsatellite instability-high; GC, gastric cancer.

Tissue microarrays (TMAs) were constructed as previously described [24]. Tissue cores (2 mm in diameter) containing two representative tumor regions–invasive front (IF) and tumor center (TC)–were taken from individual donor blocks and transferred to new recipient blocks using a trephine. Based upon the results of Galon et al.’s study of CRCs in which the densities of immune cells at the IF and TC were correlated with patient outcome [25], combined analysis of both IF and TC regions was thought to be necessary to achieve a more accurate evaluation of the prognostic significance of the tumor-associated immune cells [26,27]. One tissue core were taken at the TC and one at the IF, and TMA blocks from 143 cases were constructed (Fig 1).

Using 4-μm thick TMA tissue sections, immunohistochemical staining for CD68 and CD163 was carried out. As previously mentioned, CD68 was used as an overall infiltrated TAM marker and CD163 as an M2 macrophage marker [28]. Formalin-fixed paraffin-embedded sections were deparaffinized in xylene, rehydrated using graded alcohol. Sections were subjected to antigen retrieval using Bond-Max automated immunostainer (Leica Microsystems, Newcastle, UK). Immunohistochemical staining for each marker was conducted using the BenchMark XT immunostainer (Ventana Medical Systems, Tucson, AZ, USA) under the following conditions: CD68 (EBM11, 1:100; Dako, Glostrup, Denmark) and CD163 (10D6, 1:100; Novocastra Lab, Newcastle, UK). Stained slides were subjected to counterstaining using hematoxylin for better visualization of the tissue morphology. Staining was optimized using alveolar macrophages of lung and germinal center macrophages in the tonsil as positive control for both markers–CD68 & CD163. In addition, sections of TMA blocks were immunostained for CD8 (cytotoxic T cell) (SP16, 1:100; Neomarkers, Fremont, CA) and FoxP3 (regulatory T cell) (236A/E7, 1:100; Abcam, Cambridge, UK) for further evaluation of the correlation between tumor infiltrating lymphocytes (TILs) and TAMs. And immunohistochemical staining of *MLH1* (M1, Ventana Medical Systems, Tucson, AZ, USA), *MSH2* (FE11, 1:200, Invitrogen, Camarillo, CA, USA) were also carried out.

### Quantification of TAMs by computerized analysis

**Fig 2:**
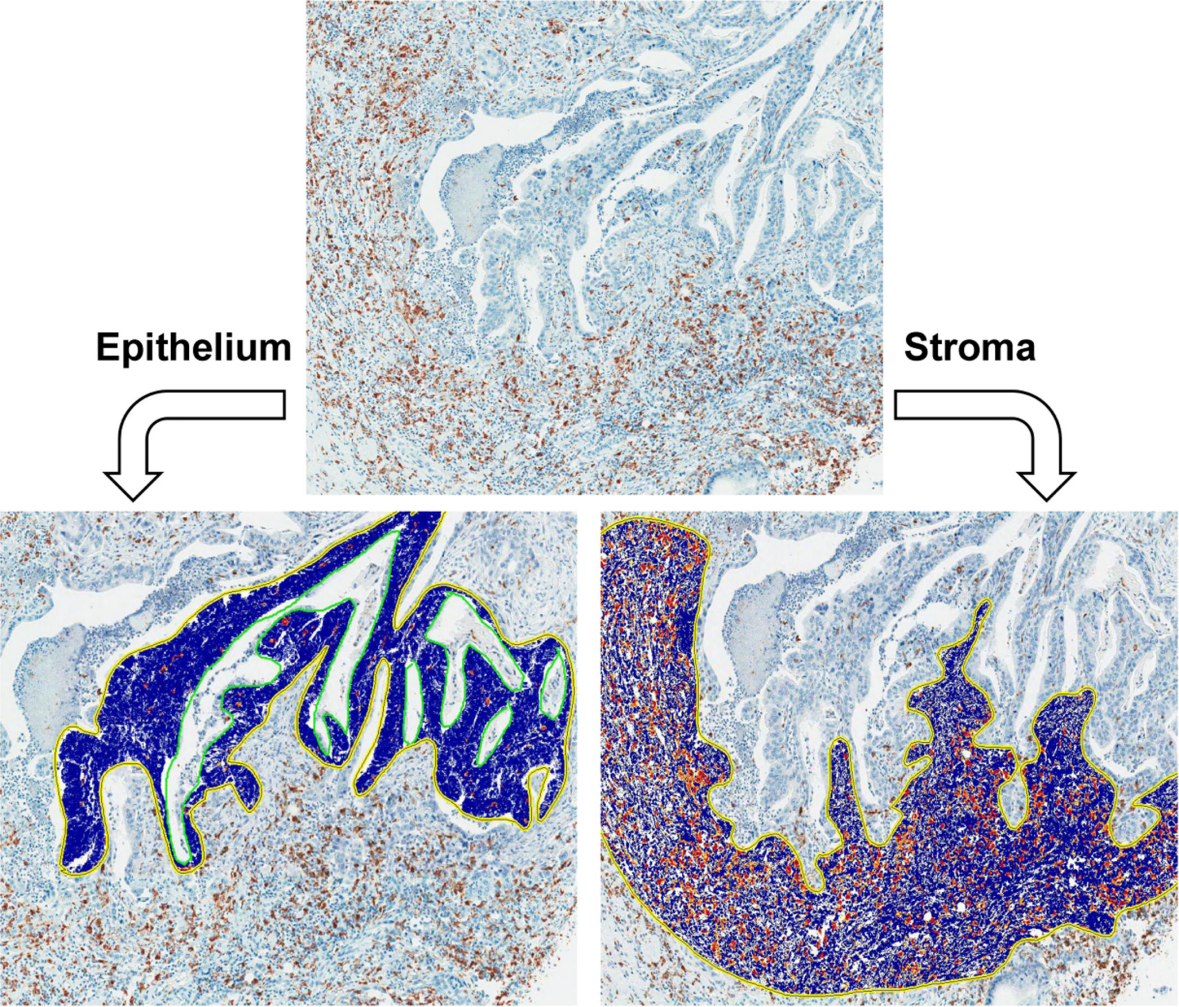
Immunohistochemical staining of CD68 and CD163 and measurement of the density of CD68+ and CD163+ TAMs. Using the automatic image analysis system (ScanScope XT; Aperio) for positive pixel count v9 algorithm, the density of CD68+ or CD163+ TAM was measured separately in the epithelium (left) and stroma (right).

All immunostained TMA slides were scanned under high-power magnification (200x) using a scanner system (ScanScope XT; Aperio Technology, Vista, CA, USA). Because CD68 and CD163 immunohistochemical staining was detected on the cell membrane and cytoplasm, which have a rough contour with variable morphology, the determination of the density by automatic counting of the number of infiltrated macrophages in selected areas using a computerized system was impossible. Instead, we used the positive pixel count v9 algorithm of ImageScope software (Aperio Technology), which defined macrophage density as areas of positively stained cells divided by all selected areas (Fig 2). To validate the accuracy of this method, we manually counted the number of the infiltrated macrophages in defined areas of 20 random cases including 10 CD68- and 10 CD163-stained cases. The correlation between the manual count and the positive pixel count of macrophages in the same area of the core was evaluated using Spearman’s rho analysis. A strong positive correlation between the two values was found (Spearman’s rank correlation coefficient r = 0.821, *p* < 0.001). In this way, the positive pixel count could be used as an alternative method for the enumeration of infiltrated CD68+ and CD163+ TAMs.

**Fig 3:**
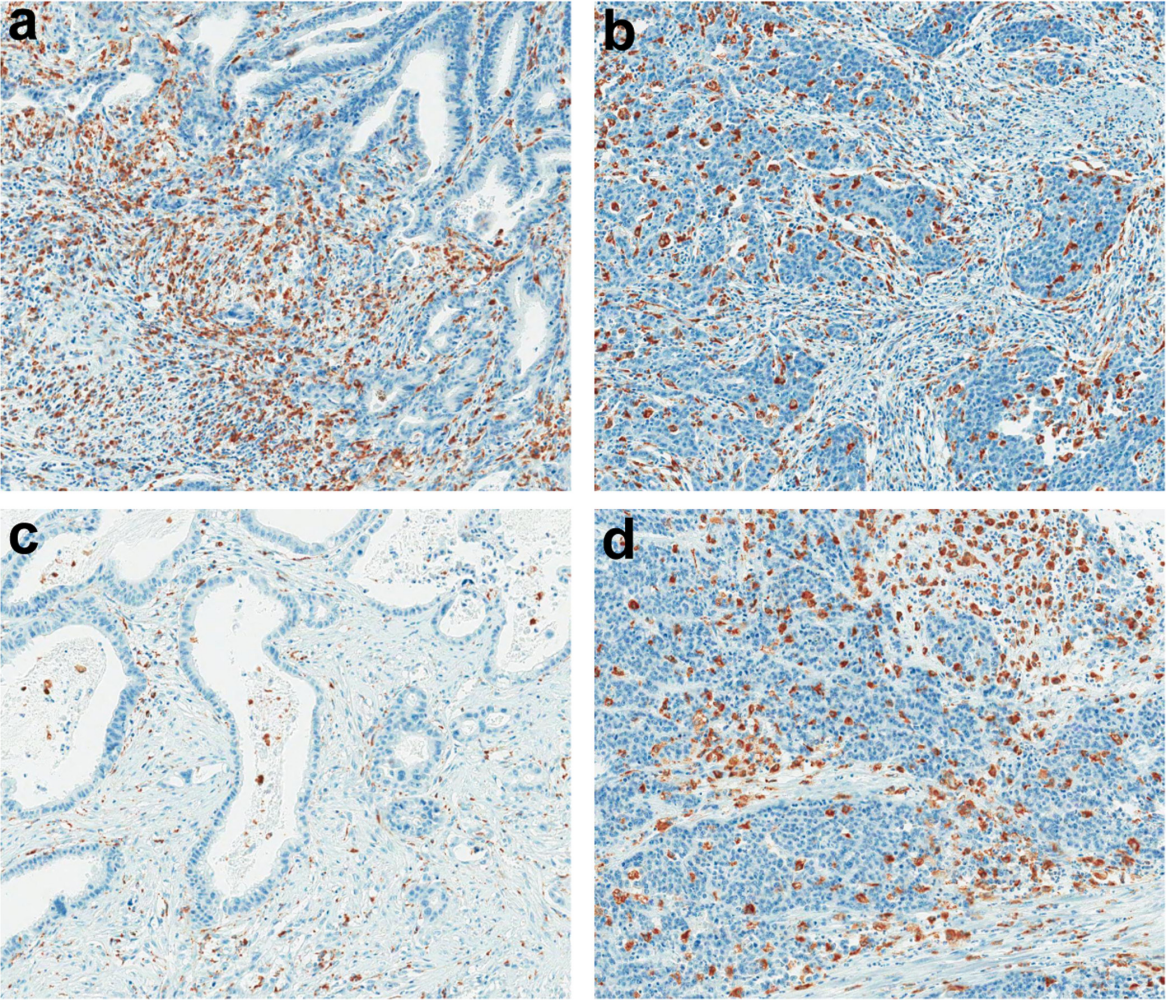
Density of CD68+ TAMs (a) stroma^high^/epithelium^low^, (b) stroma^low^/epithelium^high^, (c) stroma^low^/epithelium^low^ and (d) stroma^high^/epithelium^high^.

However, disagreement between the TAM density of the epithelial (E) and stromal (S) compartments was frequently observed. Furthermore, depending on the histologic types (intestinal, diffuse scattered and diffuse adherent), the ratio of E to S areas in the TMA cores was variable. For the reasons mentioned above, the densities of the CD68+ or CD163+ TAMs were assessed in the S and E compartments in the same core, separately, which generated densities for CD68+ or CD163+ TAMs in four different areas (S and E compartments of TC and IF regions (STC, ETC, SIF, and EIF)). The median values of the densities of CD68+ or CD163+ TAMs in each area were determined. Representative images of high and low density of TAMs in each compartment (E and S) and regions (IF and TC) is presented in Fig 3.

The densities of CD8+ and FoxP3+ TILs were analyzed according to the method described previously [24]. However, our previous study about TILs of MSI-H GCs was focused on only epithelial compartment. In this study, TILs infiltrating the stromal (S) compartment was also assessed in invasive front (IF). The density of CD8+ and FoxP3 + TILs were dichotomized into high and low density groups by using the median value.

### Determination of TAM infiltration score

**Fig 4:**
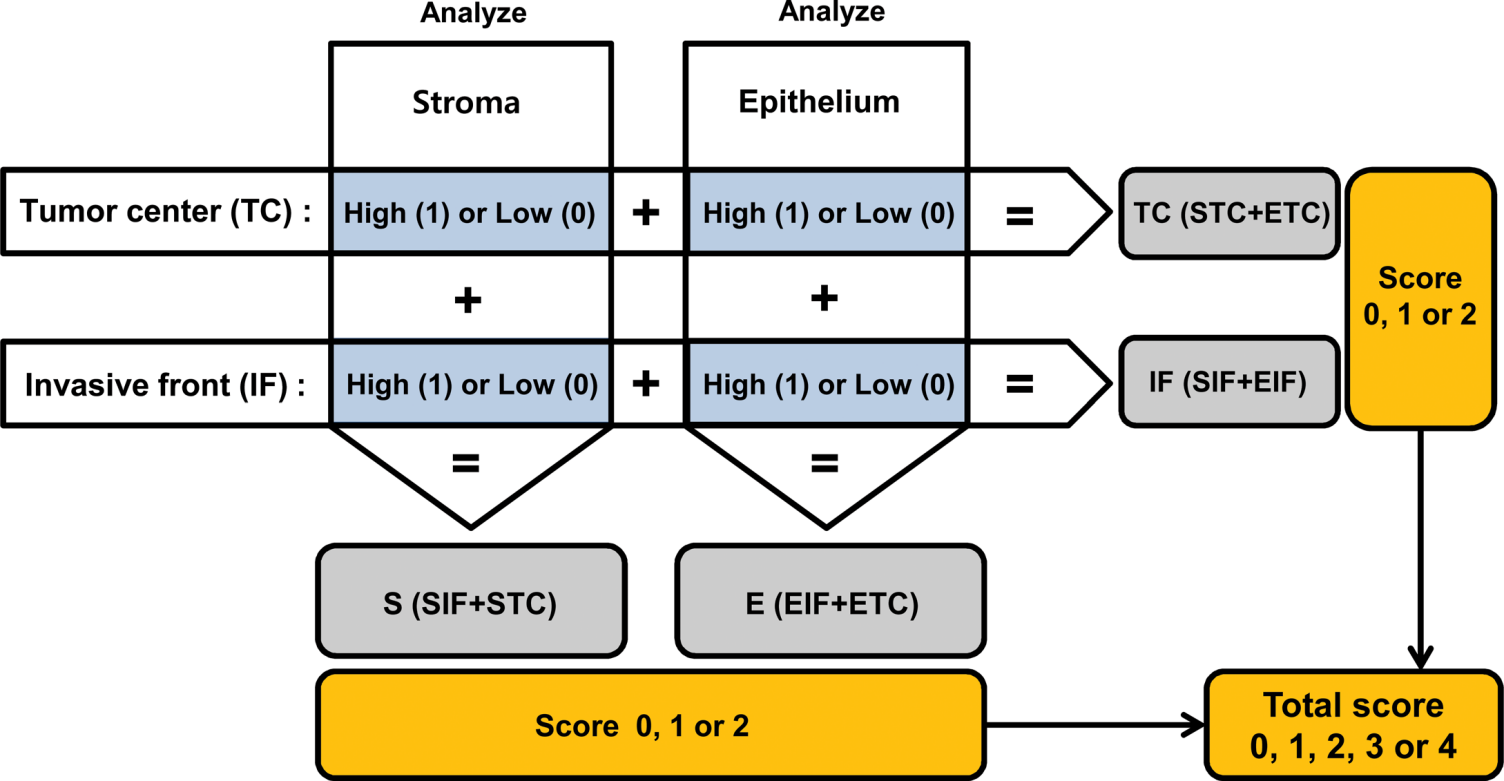
Development of the scoring system. The total score was determined by adding the scores of four different areas (S and E compartments of IF and TC regions). In addition to the total score, analysis of the compartments (S or E) and regions (IF or TC) was conducted by adding the scores of two paired areas, e.g., to determine the score of IF. We added the scores in S at IF and E at IF, which ranged from 0 to 2. For the S compartments, the scores in S at IF and S at TC were added. The same method was applied in scoring of the densities of TAMs of the other two combined areas–TC (STC + ETC) and E (EIF + ETC). *Abbreviations*: S, stroma; E, eithelium; IF, invasive front; TC, tumor center.

In the present study, for each tissue sample with TAMs, GC was scored “0” or “1” when the measured density of TAMs was below or above the median value of the respective TAM density in the specific area. With combined analysis of two or four areas, a tumor was given a sum score ranging from 0 to 2 for two areas or from 0 to 4 for four areas. Fig 4 shows how this scoring system was applied in this study. For example, in cases with CD68+ TAMs, score 4 refers to a tumor with a high density of CD68+ cells in four different areas (SIF, EIF, STC, and ETC) at the same time.

### Statistical analysis

Because the values of both TAM densities were not normally distributed based on the Kolmogorov-Smirnov test, the non-parametric correlation of TAM densities between different areas was tested using Spearman’s rank correlation test. The categorical variables were compared using Pearson's chi-square test or Fisher’s exact test (for cases with an n value <10), and the Mann-Whitney *U* test and Wilcoxon signed-rank test was used to analyze within-group differences. Kaplan-Meier survival analysis was performed to compare DFS between two subgroups according to density of CD68+ and CD163+ TAMs. Multivariate survival Cox proportional hazards regression model was used to adjust variables that may have been statistically significant for prognosis in univariate analysis. Statistical analysis was performed using the SPSS (Statistical Package for the Social Sciences) software program (version 20.0; Chicago, IL, USA). All *P* values were two sided, and *p* < 0.05 was considered statistically significant.

## Results

### Quantification analysis of CD68+ and CD163+ TAMs in MSI-H GCs

**Fig 5:**
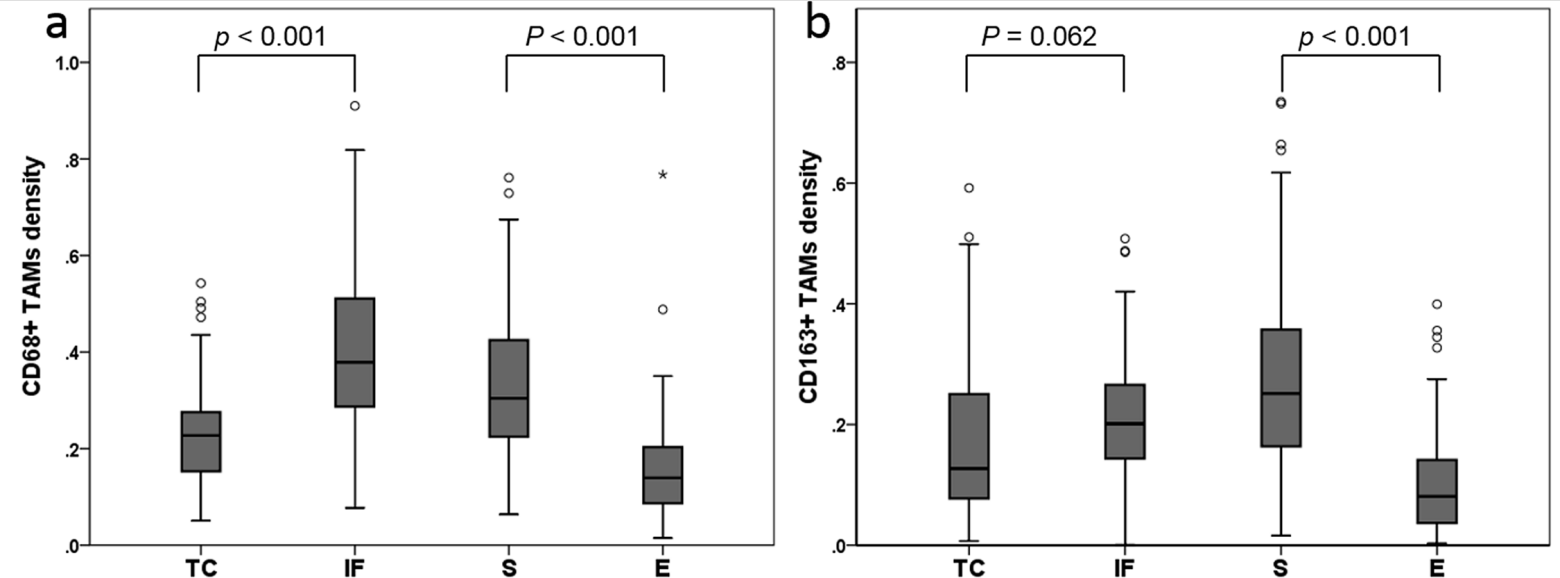
Box plots comparing the density of CD68+ or CD163+ TAMs according to different tumor areas (TC, IF, S and E) (a) The density of CD68+ TAMs was significantly higher in IF and S rather than in TC and E, respectively. (b) The density of CD163+ TAMs tends to be higher in IF than in TC and is significantly higher in S than in E. Statistical significance was evaluated using a Wilcoxon signed-rank test. *Abbreviations*: TC, tumor center; IF, invasive front; S, stroma; E, epithelium.

The distribution of positive pixel count of CD68+ and CD163+ TAMs were as follows; CD68+ TAMs in IF (SIF + EIF) (median 0.39, mean 0.401, range 0.08–0.91), TC (STC + ETC) (median 0.23, mean 0.227, range 0.05–0.54), S (SIF + STC) (median 0.30, mean 0.330, range 0.06–0.76), and E (EIF + ETC) (median 0.14, mean 0.153, range 0.01–0.77); CD163+ TAMs in IF (SIF + EIF) (median 0.20, mean 0.206, range 0.00–0.51), TC (STC + ETC) (median 0.13, mean 0.176, range 0.01–0.59), S (SIF + STC) (median 0.26, mean 0.279, range 0.02–0.73), and E (EIF + ETC) (median 0.08, mean 0.103, range 0.00–0.40). The correlations of the densities of CD68+ or CD163+ TAMs among four different areas (STC, ETC, SIF, and EIF) are summarized in S1 Table. Regardless of the type of macrophages, a positive correlation was observed among the four areas. However, between different types of macrophages, the density in SIF did not correlate with the density in the ETC. Comparison of the value of CD68+ TAMs between the IF (SIF + EIF) and TC (STC + ETC) region and between the S (SIF + STC) and E (EIF + ETC) compartments revealed a significantly higher amount of CD68+ TAMs in IF (SIF + EIF) than in TC (STC + ETC) and in S (SIF + STC) than in E (EIF + ETC) (Wilcoxon signed-rank test, *p* < 0.001 (IF vs. TC) and *p* < 0.001 (S vs. E)). For CD163+ TAMs, a similar trend for a higher infiltration of TAMs in IF (SIF + EIF) and S (SIF+ STC) compared with TC (STC + ETC) and E (EIF+ ETC) was observed (Wilcoxon signed-rank test, *p* = 0.062 (IF vs. TC) and *p* < 0.001 (S vs. E), respectively) (Fig 5).

### Association of CD68+ and CD163+ TAMs with clinicopathological features

The associations between the clinicopathological parameters of MSI-H GCs and the density of CD68+ or CD163+ TAMs in four combined areas are summarized in Table 1. Briefly, compared with the high density of CD68+ or CD163+ TAMs (sum of score = 1, 2, 3, or 4), the low density of respective one (sum of score = 0) was closely associated with more frequent low grade differentiation (well/moderately differentiated) (*p* = 0.049 and 0.004, respectively) and intestinal type histology of Lauren classification (*p* = 0.025 and 0.048, respectively). In the case of CD163+ TAMs, their low density showed a tendency to have more frequent vascular invasion (*p* = 0.124) and low BMI status (*p* = 0.077) but did not reach statistical significance. However, no significant associations were found for other parameters, including gender, age, tumor location, TNM stage, tumor depth, lymph node metastasis, presence of lymphatic or perineural invasion, Ming classification, *MLH1* expression and *MSH2* expression in CD68+ or CD163+ TAMs.

**Table 1:** Associations between CD68+ and CD163+ TAMs and the clinicopathologic characteristics in four combined area (SIF+STC+EIF+ETC).

Parameters	Case no.	CD68+ TAMs^a^			Case no.	CD163+ TAMs^a^		
		Low	High	*P* value		Low	High	*P* value
**Gender**				0.337				0.511
Male	70	16 (64.0%)	54 (53.5%)		71	16 (61.5%)	55 (52.9%)	
Female	56	9 (36.0%)	47 (46.5%)		59	10 (38.5%)	49 (47.1%)	
**Age (years)**				0.623				0.815
≤60	35	8 (32.0%)	27 (26.7%)		38	8 (30.8%)	30 (28.8%)	
>60	91	17 (68.0%)	74 (73.3%)		92	18 (69.2%)	74 (71.2%)	
**Body mass index (BMI)^b^**				0.662				0.077
Low	64	12 (48.8%)	52 (53.1%)		65	17 (68.0%)	48 (47.5%)	
High	59	13 (52.0%)	46 (46.9%)		61	8 (32.0%)	53 (52.5%)	
**Site**				0.757				0.272
Upper	9	1 (4.0%)	8 (7.9%)		9	0 (0.0%)	9 (8.7%)	
Middle	22	4 (16.0%)	18 (17.8%)		22	4 (15.4%)	18 (17.3%)	
Lower	95	20 (80.0%)	75 (74.3%)		99	22 (84.6%)	77 (74.0%)	
**AJCC stage**				0.346				1.000
I/II	83	19 (76.0%)	64 (63.4%)		86	17 (65.4%)	69 (66.3%)	
III	43	6 (24.0%)	37 (36.6%)		44	9 (34.6%)	35 (33.7%)	
**Tumor depth**				0.141				0.470
T2	35	10 (40.0%)	25 (24.8%)		37	9 (34.6%)	28 (26.9%)	
T3/T4	91	15 (60.0%)	76 (75.2%)		93	17 (65.4%)	76 (73.1%)	
**LN metastasis^b^**				0.244				1.000
Absent	81	19 (76.0%)	62 (62.0%)		83	17 (65.4%)	66 (64.1%)	
Present	44	6 (24.0%)	38 (38.0%)		46	9 (34.6%)	37 (35.8%)	
**WHO classification**				0.049				0.004
WD/MD	63	17 (68.0%)	46 (45.5%)		67	20 (76.9%)	47 (45.2%)	
PD	63	8 (32.0%)	55 (54.5%)		63	6 (23.1%)	57 (54.8%)	
**Lymphatic invasion**				0.110				0.117
Absent	49	6 (24.0%)	43 (42.6%)		48	6 (23.1%)	42 (40.4%)	
Present	77	19 (76.0%)	58 (57.4%)		82	20 (76.9%)	62 (59.6%)	
**Vascular invasion**				0.210				0.124
Absent	107	19 (76.0%)	88 (87.1%)		110	19 (73.1%)	91 (87.5%)	
Present	19	6 (24.0%)	13 (12.9%)		20	7 (26.9%)	13 (12.5%)	
**Perineural invasion**				1.000				0.642
Absent	82	16 (64.0%)	66 (65.3%)		87	16 (61.5%)	71 (68.3%)	
Present	44	9 (36.0%)	35 (34.7%)		43	10 (38.5%)	33 (31.7%)	
**Lauren classification**				0.025				0.048
Intestinal	70	19 (76.0%)	51 (50.5%)		73	19 (73.1%)	54 (51.9%)	
Diffuse	56	6 (24.0%)	50 (49.5%)		57	7 (26.9%)	50 (48.1%)	
**Ming classification**				1.000				0.460
Expanding	34	7 (28.0%)	27 (26.7%)		34	5 (19.2%)	29 (27.9%)	
Infiltrative	92	18 (72.0%)	74 (73.3%)		96	21 (80.8%)	75 (72.1%)	
***MLH1* expression^c^**				1.000				1.000
Retained	13	2 (8.7%)	11 (11.5%)		12	2 (8.0%)	10 (10.1%)	
Loss	106	21 (91.3%)	85 (88.5%)		112	23 (92.0%)	89 (89.9%)	
***MSH2* expression^c^**				1.000				0.685
Retained	110	22 (95.7%)	88 (91.7%)		115	24 (96.0%)	91 (91.9%)	
Loss	9	1 (4.3%)	8 (8.3%)		9	1 (4.0%)	8 (8.1%)	

^a^ Included only for patients with available TMA data.

^b^ Included only for patients with available clinicopathologic data.

^c^ Included only for patients with available immunohistochemistry data.

TAM, tumor associated macrophage; SIF, stromal tumor associated macrophage density in invasive front; STC, stromal tumor associated macrophage density in tumor center; EIF, epithelial tumor associated macrophage density in invasive front; ETC, epithelial tumor associated macrophage density in tumor center; LN, lymph node; BMI, body mass index

In addition, the correlation between the clinicopathologic parameters and the density of CD68+ or CD163+ TAMs was also analyzed in two combined areas (IF (SIF + EIF) (S2 Table), TC (STC + ETC) (S3 Table), S (SIF + STC) (S4 Table), and E (EIF + ETC) (S5 Table). Similarly to the previous results in four combined areas, an association of high TAM density with high grade tumor differentiation and diffuse type histology of Lauren classification was found in IF (SIF + EIF) and E (EIF + ETC). However, in the S compartments (SIF + STC), none of the analyzed parameters was significantly correlated with each TAM density. Moreover, in the TC (STC + ETC) region, the low density of CD68+ TAMs was significantly correlated with more frequent lymphatic invasion (*p* = 0.046). In the E compartments (EIF + ETC), the low density of CD68+ TAMs was associated with a more frequent of low grade differentiation (*p* <0.001) and a higher frequency of vascular invasion (*p* = 0.042), and the low density of CD163+ TAMs was linked with more frequent lymphatic invasion (*p* = 0.044).

### Survival analysis

**Fig 6:**
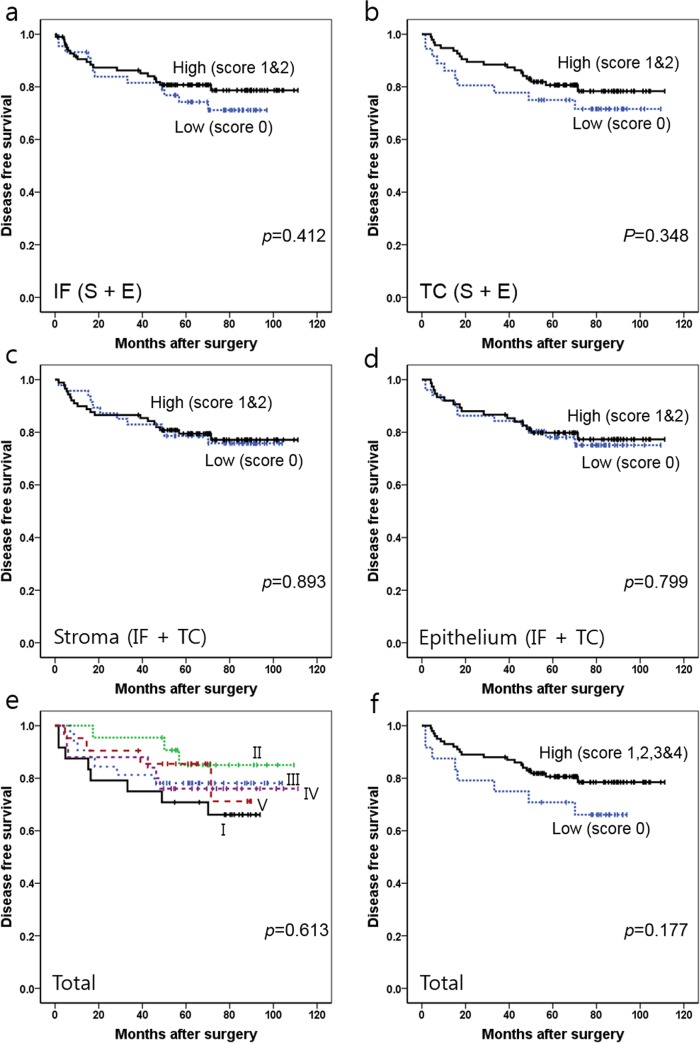
Kaplan-Meier survival analysis with log-rank test of CD68+ TAMs. (a) Survival curves of the low density (score 0) (n = 44) vs. high density (score 1 and 2) (n = 98) groups in IF (SIF + EIF). (b) Survival curves of the low density (score 0) (n = 36) vs. high density (score 1 and 2) (n = 95) groups in TC (STC + ETC). (c) Survival curves of the low density (score 0) (n = 47) vs. high density (score 1 and 2) (n = 89) groups in S (SIF+STC). (d) Survival curves of the low density (score 0) (n = 51) vs. high density (score 1 and 2) (n = 75) groups in E (EIF+ETC). (e) Survival curves of five subgroups determined by the total score in four combined areas (SIF + STC + EIF + ETC) (I, score 0 (n = 24); II, score 1 (n = 22); III, score 2 (n = 32); IV, score 3 (n = 25) and V, score 4 (n = 21)). (f) Survival curves of the low density (score 0) (n = 24) vs. high density (score 1–4) (n = 100) groups in four combined areas (SIF + STC + EIF + ETC). *Abbreviations*: TC, tumor center; IF, invasive front; S, stroma; E, epithelium.

The average DFS was 62 months (ranging from 2 to 111 months). For CD68+ TAMs (Fig 6), patients with a high infiltration (sum score = 1 or 2) in the IF region (SIF + EIF) showed a tendency toward better DFS than those with a low infiltration (sum score = 0) in the IF region (SIF + EIF), but statistical significance was not reached (*p* = 0.412). A similar trend was observed for survival analysis in the TC region (STC + ETC) (*p* = 0.348). However, when DFS was compared according to the density of CD68+ TAMs in the S compartments (STC + SIF), no significant survival difference was observed between groups with high (sum score = 1 or 2) and low density (sum score = 0) (*p* = 0.893). No difference was also noted in DFS between high (sum score = 1 or 2) and low density (sum score = 0) in the E compartments (ETC + EIF) (*p* = 0.799). When we combined the analysis of four different areas (SIF, STC, EIF, and ETC), the prognostic difference was obscure between five subgroups that scored from 0 to 4 (*p* = 0.613). However, the survival curves essentially clustered into two subgroups (low density (sum score = 0) and high density (sum score = 1, 2, 3, or 4), with a tendency for prolonged DFS in the high density group (*p* = 0.177). A similar trend in each region and compartment was found when we performed survival analysis excluding stage I cases (n = 26) (S1 Fig).

**Fig 7:**
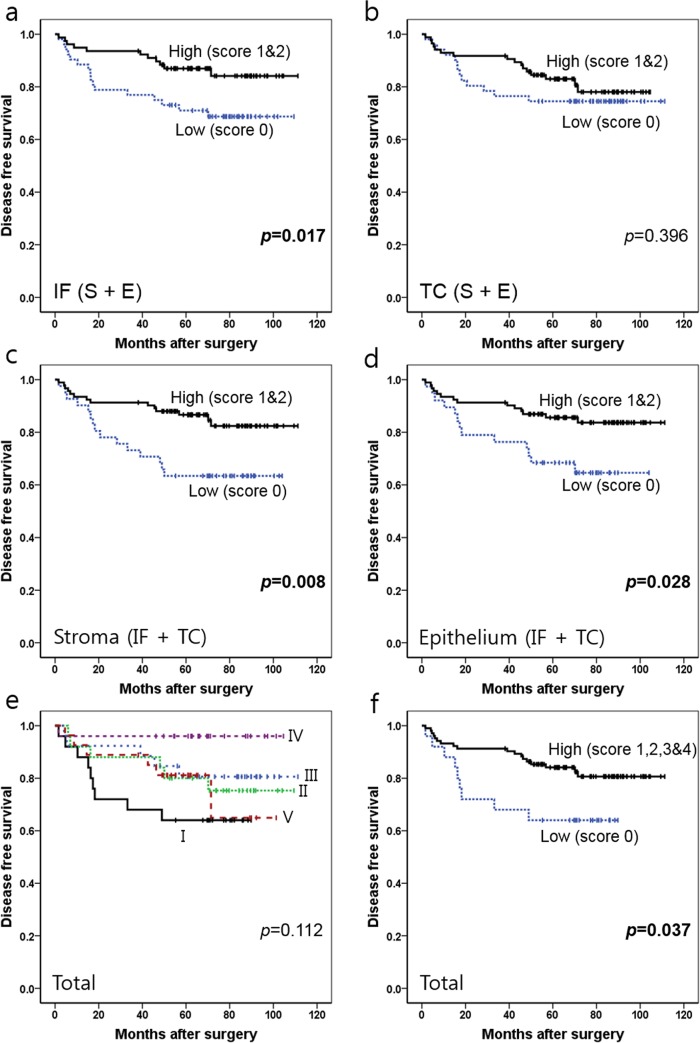
Kaplan-Meier survival analysis with log-rank test of CD163+ TAMs. (a) Survival curves of the low density (score 0) (n = 38) vs. high density (score 1 and 2) (n = 92) groups in IF (SIF + EIF). (b) Survival curves of the low density (score 0) (n = 51) vs. high density (score 1 and 2) (n = 85) groups in TC (STC + ETC). (c) Survival curves of the low density (score 0) (n = 41) vs. high density (score 1 and 2) (n = 92) groups in S (SIF+STC). (d) Survival curves of the low density (score 0) (n = 52) vs. high density (score 1 and 2) (n = 78) groups in E (EIF+ETC). (e) Survival curves of the five subgroups determined by the total score in four combined areas (SIF + STC + EIF + ETC) (I, score 0 (n = 25); II, score 1 (n = 25); III, score 2 (n = 26); IV, score 3 (n = 25) and V, score 4 (n = 27)). (f) Survival curves of the low density (score 0) (n = 25) vs. high density (score 1–4) (n = 103) groups in four combined areas (SIF + STC + EIF + ETC). *Abbreviations*: TC, tumor center; IF, invasive front; S, stroma; E, epithelium.

For CD163+ TAMs, a close association between high density and better survival was found in the IF region (SIF + EIF), S compartments (SIF + STC), and E compartments (EIF + ETC), but not in the TC region (STC + ETC) (Fig 7). Patients with a high density (sum score = 1 or 2) in the IF region (SIF + EIF) exhibited better DFS (*p* = 0.017) than patients with a low density (sum score = 0) in the IF region (SIF + EIF). However, the prognostic value of the density of CD163+ TAMs in the TC region (STC + ETC) did not reach statistical significance (*p* = 0.396). When the prognosis was compared in each compartment, a significant DFS advantage was observed in patients with a high density of CD163+ TAMs (sum score = 1 or 2) in the S compartments (SIF + STC) (*p* = 0.008) compared with those with a low density (sum score = 0). A similar tendency was found in the E compartments (EIF + ETC) (*p* = 0.028). With combined analysis of CD163+ TAMs in four areas (SIF, STC, EIF, and ETC), a subgroup with sum score 0 exhibited the worst DFS and subgroup with score 4 showed the second worst DFS (*p* = 0.112) without linear trend. When analysis was performed after combining subgroups with sum-score ranging from 1 to 4 into one group, score 0 group exhibited significantly worse DFS (*p* = 0.037). When we excluded the stage I cases, a high density of CD163+ TAM showed better prognosis in IF (*p* = 0.012), S (*p* = 0.002), E (*p* = 0.035) and four-combined area (*p* = 0.006) but not in TC region (*p* = 0.251) (S2 Fig). To sum up the above data, high density of CD163+ TAMs was significantly associated with prolonged DFS in MSI-H GCs, but in the cases of CD68+ TAMs, high density was marginally significant in predicting patients’ good clinical outcome.

To further evaluate the prognostic implication of CD68+ or CD163+ TAMs in MSI-H GCs, univariate and multivariate analysis using a Cox proportional hazard regression model was performed. In univariate analysis, high TNM stage, presence of lymphatic, vascular and perineural invasion, and infiltrative type by Ming classification were significantly associated with reduced DFS (Table 2). With these variables, which have a significant prognostic effect in univariate analysis as listed, the categorical variables of the density of CD163+ TAMs according to regions and compartments were added one by one in multivariate analysis. After adjusting for possible confounding factors, high density of CD163+ TAMs in the four combined areas (STC + ETC + SIF+ EIF) was observed to be an independent good prognostic indicator (*p* = 0.030) (Table 2). High density of CD163+ TAMs in the S (SIF + STC) and E (EIF + ETC) were also statistically significant factors that predicted good survival outcome (*p* = 0.028 (S), 0.048 (E)) (Table 3).

**Table 2:** Univariate and multivariate survival analysis of factors associated with progression-free survival.

		Univariate analysis		Multivariate analysis	
Parameters	n	Mean survival time	*P* value	Hazard ratio	*P* value
		(month; 95% confidence interval)		(month; 95% confidence interval)	
**Gender**			0.896	(-)	
Male	62	65 (58–71)			
Female	77	58 (51–65)			
**Age (years)**			0.371	(-)	
≤60	41	58 (48–67)			
>60	98	64 (58–69)			
**Body mass index (BMI)^a^**			0.626	(-)	
Low	67	62 (54–69)			
High	62	64 (57–70)			
**AJCC stage**			<0.001		<0.001
I/II	92	72 (68–77)		Reference	
III	47	50 (37–55)		6.917 (2.602–18.390)	
**Site**			0.487	(-)	
Upper	11	51 (33–67)			
Middle	21	56 (41–71)			
Lower	107	64 (59–70)			
**Lymphatic invasion**			0.024		0.456
Absent	51	68 (63–74)		Reference	
Present	88	59 (52–65)		1.552 (0.488–4.933)	
**Vascular invasion**			0.027		0.249
Absent	120	65 (60–69)		Reference	
Present	19	47 (30–65)		1.804 (0.662–4.914)	
**Perineural invasion**			0.010		0.795
Absent	91	67 (60–72)		Reference	
Present	48	53 (44–63)		1.116 (0.489–2.544)	
**Ming classification**			0.029		0.056
Expanding	37	78 (70–85)		Reference	
Infiltrative	102	57 (51–62)		7.101 (0.949–53.117)	
**Lauren classification**			0.969	(-)	
Intestinal	77	65 (59–72)			
Diffuse	62	57 (50–64)			
**CD68+ TAM density–total^b^**			0.156	(-)	
Low (score 0)	24	63 (49–76)			
High (score 1–4)	99	65 (59–70)			
**CD163+ TAM density–total^b^**			0.035		0.030
Low (score 0)	25	56 (44–69)		Reference	
High (score 1–4)	102	68 (63–73)		0.386 (0.163–0.910)	
**CD163+ TAM density–IF^b^(SIF+EIF)**			0.015	(-)	
Low (score 0)	38	58 (49–67)			
High (score 1,2)	91	69 (64–75)			
**CD163+ TAM density–S^b^(SIF+STC)**			0.007	(-)	
Low (score 0)	41	61 (51–71)			
High (score 1,2)	91	67 (62–72)			
**CD163+ TAM density–E^b^(EIF+ETC)**			0.049	(-)	
Low (score 0)	51	63 (54–72)			
High (score 1,2)	78	68 (62–73)			
**FoxP3+ TILs density–IF(E+S)**			0.001	(-)	
Low	72	57 (49–65)			
High	71	65 (60–71)			
**CD8+ TILs density–IF(E+S)**			0.005	(-)	
Low	70	59 (51–67)			
High	73	63 (58–69)			

^a^ Included only for patients with available clinicopathologis data.

^b^ Included only for patients with available TMA data.

TAM, tumor associated macrophage; SIF, stromal tumor associated macrophage density in invasive front; STC, stromal tumor associated macrophage density in tumor center; EIF. epithelial tumor associated macrophage density in invasive front; ETC, epithelial tumor associated macrophage density in tumor center; IF, invasive front; E, epithelium; S, stroma; BMI, body mass index

**Table 3:** Multivariate survival analysis of factors associated with progression-free survival.

	Multivariate analysis—II		Multivariate analysis—III		Multivariate analysis—IV	
Parameters	Hazard ratio	*P* value	Hazard ratio	*P* value	Hazard ratio	*P* value
	(month;95% confidence interval)		(month;95% confidence interval)		(month;95% confidence interval)	
**AJCC stage**		<0.001		<0.001		<0.001
I/II	Reference		Reference		Reference	
III	6.112 (2.340–15.966)		5.410 (2.185–13.395)		6.634 (2.513–17.513)	
**Ming classification**		0.065		0.059		0.042
Expanding	Reference		Reference		Reference	
Infiltrative	6.677 (0.886–20.290)		7.003 (0.928–52.818)		8.097 (1.078–60.786)	
**Lymphatic invasion**		0.316		0.449		0.585
Absent	Reference		Reference		Reference	
Present	1.770 (0.580–53.401)		1.483 (0.535–4.111)		1.395 (0.423–4.597)	
**Vascular invasion**		0.105		0.085		0.179
Absent	Reference		Reference		Reference	
Present	2.256 (0.844–6.030)		2.340 (0.890–6.150)		1.967 (0.733–5.279)	
**Perineural invasion**		0.850		0.993		0.657
Absent	Reference		Reference		Reference	
Present	1.083 (0.474–2.477)		1.003 (0.443–2.274)		1.207 (0.526–2.768)	
**CD163+ TAM density–IF(SIF+EIF)**		0.051		Not included*		Not included*
Low (score 0)	Reference					
High (score 1,2)	0.458 (0.209–1.004)					
**CD163+ TAM density–S(SIF+STC)**		Not included*		0.028		Not included*
Low (score 0)			Reference			
High (score 1,2)			0.413 (0.188–0.910)			
**CD163+ TAM density–E(EIF+ETC)**		Not included*		Not included*		0.048
Low (score 0)					Reference	
High (score 1,2)					0.428 (0.185–0.991)	

* Because the covariate associated with CD163+ tumor associated macrophage density–IF (SIF+EIF), S (SIF+STC) and E (EIF+ETC)–are positively associated with each other, they could not be included in multivariate analysis at the same time. Therefore, these parameters were added to each set of multivariate analysis one by one.

TAM, tumor associated macrophage; SIF, stromal tumor associated macrophage density in invasive front; STC, stromal tumor associated macrophage density in tumor center; EIF, epithelial tumor associated macrophage density in invasive front; ETC, epithelial tumor associated macrophage density in tumor center; IF, invasive front; E, epithelium; S, stroma; BMI, body mass index.

### Association between densities of TILs and CD163+TAMs

**Fig 8:**
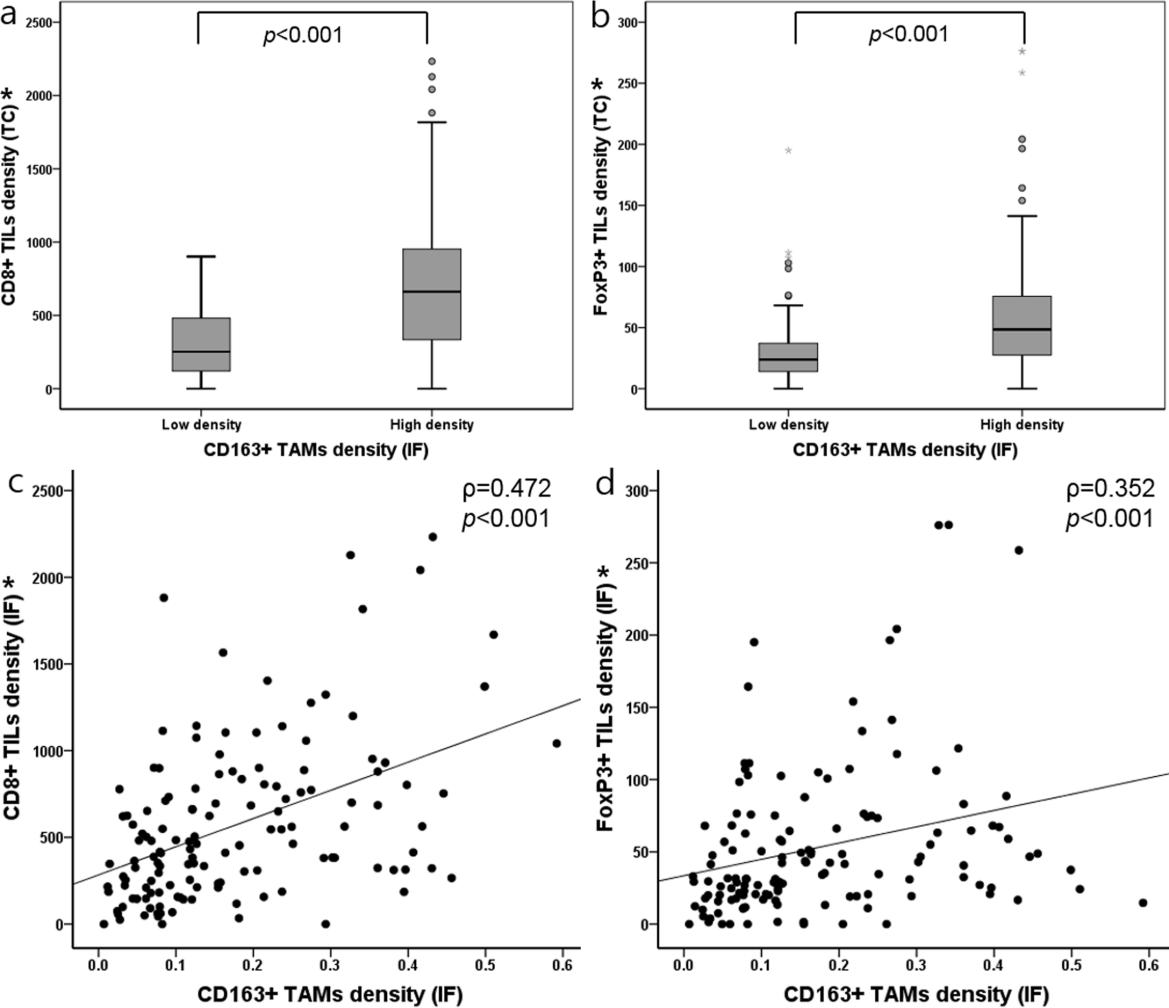
(a, b) Box plots illustrating densities of CD8+ or FoxP3+ TILs in dependence of CD163+ TAMs density in IF regions (Mann-Whitney *U* test). (c, d) Association between the density of CD8+ or FoxP3 TILs and the density of CD163+ TAMs (Spearman’s rank correlation test). * The density of CD8+ and FoxP3+ TILs is shown as the number of infiltrated lymphocytes per unit area (mm^2^) in IF irrespective of S or E compartment. *Abbreviations*: TIL, tumor infiltrating lymphocyte; IF, invasive front; S, stroma; E, epithelium.

When we searched for correlation between TAMs and TILs in MSI-H GCs, we found that densities of CD8+ and FoxP3+ TILs in IF region were higher in high CD163+ TAM density group compared to low CD163+ TAM density group (*p* < 0.001) (Mann-Whitney *U* test) (Fig 8). Indeed, the densities of CD8+ and FoxP3+ TILs were positively correlated with the density of CD163+ TAMs (*p* < 0.001) (Spearman’s rank correlation test) (Fig 8).

**Fig 9:**
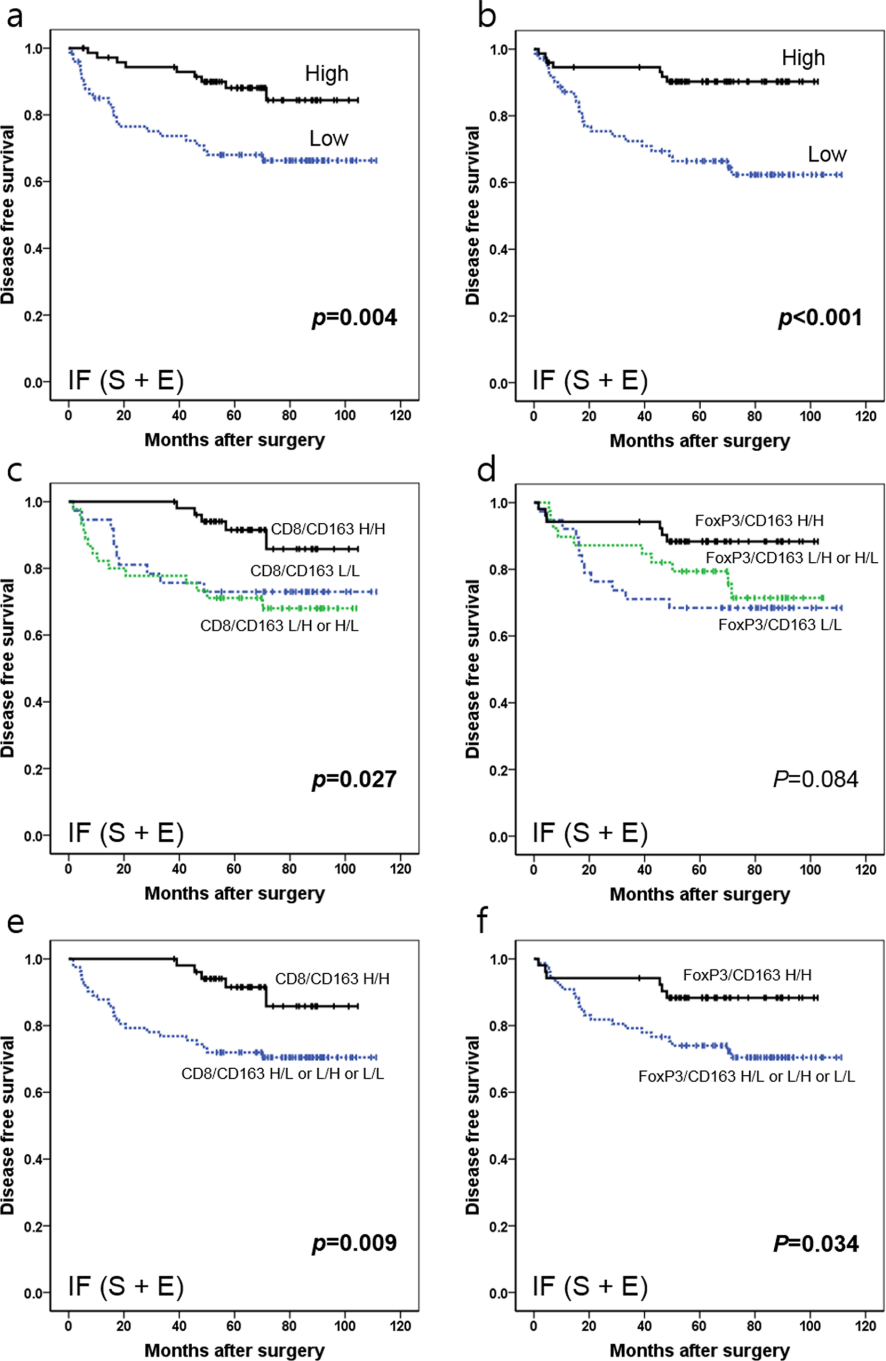
Kaplan-Meier survival analysis with log-rank test according to combinatory statuses of CD8+ or FoxP3+ TILs and CD163+ TAMs. (a) Survival curves of the low density (n = 70) vs. high density (n = 73) groups of CD8+ TILs in IF. (b) Survival curves of the low density (n = 72) vs. high density (n = 71) groups of FoxP3+ TILs in IF. (c) Survival curves of CD8+^high^ /CD163+^high^ (n = 52) vs. CD8+^high^ / CD163+^low^ or CD8+^low^ /CD163+^high^ (n = 45) vs. CD8+^low^ /CD163+^low^ (n = 37) (d) Survival curves of FoxP3+^high^ /CD163+^high^ (n = 52) vs. FoxP3+^high^ / CD163+^low^ or FoxP3+^low^ /CD163+^high^ (n = 39) vs. FoxP3+^low^ /CD163+^low^ (n = 38). *Abbreviations*: TIL, tumor infiltrating lymphocyte; TC, tumor center; IF, invasive front; S, stroma; E, epithelium; H, high density; L, low density.

Kaplan-Meier survival analysis showed a significant advantage of DFS in cases with high density of CD8+ or FoxP3+ TIL compared to cases with a low density of corresponding TILs (*p* = 0.004 and 0.001, respectively) (Fig 9). With combination of TIL and TAM variables, the CD8+^high^/CD163+^high^ and FoxP3+^high^/CD163+^high^ group showed the best DFS (*p* = 0.009 and 0.034, respectively) compared with other groups (Fig 9).

## Discussion

In the present study, we analyzed the prognostic implication of CD68+ or CD163+ TAMs in MSI-H advanced GCs. In the tumor microenvironment, macrophages constitute a major component of tumor-infiltrating leukocytes and affect tumor cells by releasing many chemical substances. Two phenotypic subtypes of TAMs–M1 and M2 macrophages–have been reported to have opposite roles in tumor progression. M2 macrophages are thought to have tumor promoting functions whereas M1 macrophages have shown a protective role in tumorigenesis. As TAMs are more closely linked to M2 type rather than M1 type macrophages, many studies have demonstrated that high levels of TAMs are associated with poor clinical outcome in human cancers, including breast, ovary, lung and endometrial cancers [9, 12–15]. However, in GCs, conflicting results have been reported [29].

CD68 has been used as a marker of overall infiltrated TAM covering a majority of functionally activated macrophages regardless of their polarization state in many studies. Wang et al.’s study showed that intra-tumoral infiltrating CD68+ TAMs are independent good prognostic factors in GCs [21]. In contrast, Wu et al.’s study demonstrated that CD68+ TAMs promote angiogenesis and lymphangiogenesis of GCs [30]. Additionally, Ishigami et al. argued that patients with a high count of CD68+ TAMs had poorer surgical outcomes than those with a low count [31]. However, Zhang et al.’s study showed that CD68+ TAMs in GCs have no significant association with overall survival [32]. In our study, CD68+ TAMs were found to have no prognostic impact on DFS in MSI-H advanced GCs. Along with CD68+ TAMs, recent studies have focused on the specific role of each subset of TAMs by discriminating the M1 and M2 phenotypes.

CD163 is a member of histiocyte/macrophage-associated scavenger receptor [33]. Although the expression of CD163 has been reported in cells other than M2 macrophages, such as dendritic cells, CD163 can be a useful marker to distinguish M2 macrophages from other subsets. Many studies have demonstrated an adverse prognostic effect of M2 macrophages on clinical outcome in GCs [32, 34]. However, a beneficial role of M2 macrophages on prognosis has been reported in hollow viscus tumors, e.g., CRCs. Algar et al.’s study concluded that the type and location of tumor infiltrating macrophages contribute to the clinical behavior of CRCs in a stage-specific manner, by demonstrating that in stage III CRCs, a high number of M2 macrophages in the peritumoral area correlated with prolonged cancer-specific survival time [35]. However, in a more advanced stage, a reverse correlation was observed. In addition, Edin et al.’s study also showed that increased infiltration of M1 or M2 type macrophages at the invasive front was associated with significantly improved cancer-related survival in CRCs [18]. Consistent with this report, our findings demonstrated that high density of CD163+ TAMs is an independent prognostic indicator heralding better prognosis in MSI-H advanced GCs.

The mechanism of our data that challenge the current theory regarding the prognostic role of CD163+ TAMs has not been entirely elucidated. However, several authors have argued that M2 macrophages have less deleterious effects in CRCs, which is attributed to the unique intestinal environment with numerous colonizing micro-organisms [18, 35, 36]. This less hazardous effect of M2 macrophages can be applied to GCs considering the fact that the vast majority of GCs arise in the stomach infected with *Helicobacter pylori* and that GCs have molecular mechanisms of carcinogenesis in common with CRCs. In addition, the exaggerated number of TILs which occur as a consequence of MSI seems to exert considerable influence on prognostic role of CD163+ TAMs in MSI-H GCs. In our survival analysis according to combinatory statuses of TIL and TAM, tumors with high density of CD163+ TAMs plus high density of CD8+ TILs or high density of FoxP3+ TILs showed better survival compared with 1) tumors with high density of CD163+ TAMs plus low density of CD8+ or FoxP3+ TILs or 2) tumors with low density of CD163+ TAMs plus high density of CD8+ or FoxP3+ TILs. These finding means that high density of CD163+ TAMs only could not guarantee the patient’s favorable outcome without accompanying existence of high density of TILs at the same time. Based on these findings, TAMs and TILs are considered to interact on each other and the prognostic effect can be determined in proportional balance of both TAMs and TILs in MSI-H GCs. The parallel analysis in other molecular subtypes, including MSI- and EBV-negative GCs or EBV-positive GCs, is required for figuring out the prognostic role of balanced high infiltrations of both TAMs and TILs and the exact role of MSI on functional differentiation of CD163+ TAMs. Moreover, M2 macrophages are thought to be involved in immunoregulation by inducing the skewing of TILs toward a more regulatory phenotype [7, 37] which have protective role on tumor development in MSI-H GCs according to our previous study [24].

In the past, studies of the prognostic impact of tumor infiltrating immune cells have primarily focused on immune cells in the invasive front [20]. However, combined analysis of immune cells in both the TC and IF regions has been demonstrated to have a prognostic significance superior to single region analysis [26, 27]. Furthermore, because a considerable number of TAMs also exist in the E compartment along with the S compartment and the number of infiltrated TAMs was not always directly proportional between the E and S compartments, we performed a numerical quantification of TAMs in the E and S compartments of the tumor regions separately. Thus, in the present study, we assessed the density of CD68+ or CD163+ TAMs in four separate areas (STC, ETC, SIF, and EIF) and analyzed the prognostic value of intratumoral CD68+ or CD163+ TAMs in terms of their density and location. Our findings suggest that the intratumoral density of CD163+ TAMs affects the clinical behavior of MSI-H GCs but that of CD68+ TAMs does not. Combined assessment of CD163+ TAMs in the S compartments (SIF + STC) was found to be superior to assessments of CD163+ TAMs in EIF + ETC, EIF + SIF, ETC + STC or ETC + STC + EIF + SIF in survival analysis.

We initially attempted to use computerized image analysis but realized that accurate automatic counting of macrophages was very challenging due to highly variable shape and size of macrophages. Therefore, the positive pixel count v9 algorithm of Aperio analysis was applied, which measured the total positively stained cellular area in all selected area. This analysis was shown to be a valid alternative method by comparing to manual counting data in 20 random cases. Compared with a previous study using a qualitative grading system, this method is more objective and reproducible. To the best of our knowledge, our study is the first attempt to analyze TAMs by not only dividing the tumor tissues into four different areas but also using quantitative computerized image analysis in MSI-H GCs.

Several limitations exist in the present study. First, we could not analyze M1 macrophages because appropriate immunohistochemical antibodies specific for M1 type macrophages were not available. According to previous studies of CRCs, it has been hypothesized that M1 phenotype macrophages primarily function in the tumor microenvironment associated with M2 macrophage’s less hazardous tumor progression effect [19,38,39]. The prognostic effect of M1 macrophages should be addressed in future studies. Second, not all CD163+ stained cells are M2 macrophages, and small portions of positively stained cells belong to other subsets such as dendritic cells [33]. If double immunohistochemical staining of CD68 and C163 had been carried out, a more refined characterization of M2 macrophages may be obtained.

Recently, growing evidence regarding the role of TAMs in tumor progression has led to new therapeutic options targeting TAMs. The main approaches of this immunotherapy include the depletion of TAMs, reprogramming of TAMs from the M2 to M1 phenotype or the inhibition of TAMs in the tumor microenvironment [7, 40]. However, in our study, we demonstrate that CD163+ TAMS have a beneficial effect on survival in MSI-H GCs, raising concern that approaches directed against M2 TAMs might exacerbate the course of the disease of patients with MSI-H GC. Follow-up studies addressing the influence of TAMs on tumor progression in MSI-H GCs are encouraged to develop therapeutic strategies appropriate for GCs.

In summary, our study demonstrated that a high density of CD163+ TAMs is an independent determinant of good clinical outcome in patients with MSI-H GCs and that the combined analysis of CD163+ TAMs in the S compartments of both the TC and IF regions might serve as a useful prognostic parameter in MSI-H GCs.

## Supporting Information

S1 Fig Kaplan-Meier survival analysis with log-rank test of CD68+ TAMs in stage II and stage III. (a) Survival curves of the low density (score 0) (n = 32) vs. high density (score 1 and 2) (n = 87) groups in IF (SIF + EIF). (b) Survival curves of the low density (score 0) (n = 26) vs. high density (score 1 and 2) (n = 82) groups in TC (STC + ETC). (c) Survival curves of the low density (score 0) (n = 38) vs. high density (score 1 and 2) (n = 74) groups in S (SIF+STC). (d) Survival curves of the low density (score 0) (n = 36) vs. high density (score 1 and 2) (n = 68) groups in E (EIF+ETC). (e) Survival curves of five subgroups determined by the total score in four combined areas (SIF + STC + EIF + ETC) (I, score 0 (n = 16); II, score 1 (n = 16); III, score 2 (n = 29); IV, score 3 (n = 24) and V, score 4 (n = 17)). (f) Survival curves of the low density (score 0) (n = 16) vs. high density (score 1–4) (n = 86) groups in four combined areas (SIF + STC + EIF + ETC). *Abbreviations*: TC, tumor center; IF, invasive front; S, stroma; E, epithelium.
(TIF)

S2 Fig Kaplan-Meier survival analysis with log-rank test of CD163+ TAMs in stage II and stage III. (a) Survival curves of the low density (score 0) (n = 30) vs. high density (score 1 and 2) (n = 77) groups in IF (SIF + EIF). (b) Survival curves of the low density (score 0) (n = 39) vs. high density (score 1 and 2) (n = 73) groups in TC (STC + ETC). (c) Survival curves of the low density (score 0) (n = 34) vs. high density (score 1 and 2) (n = 76) groups in S (SIF+STC). (d) Survival curves of the low density (score 0) (n = 37) vs. high density (score 1 and 2) (n = 70) groups in E (EIF+ETC). (e) Survival curves of five subgroups determined by the total score in four combined areas (SIF + STC + EIF + ETC) (I, score 0 (n = 18); II, score 1 (n = 19); III, score 2 (n = 24); IV, score 3 (n = 22) and V, score 4 (n = 22)). (f) Survival curves of the low density (score 0) (n = 18) vs. high density (score 1–4) (n = 87) groups in four combined areas (SIF + STC + EIF + ETC). *Abbreviations*: TC, tumor center; IF, invasive front; S, stroma; E, epithelium.
(TIF)

S1 Table Correlation of CD68+ and CD163+ TAMs density according to each compartment in each region in MSI-H GCs. (DOCX)

S2 Table Associations between CD68+ and CD163+ TAMs with clinicopathologic characteristics in IF (SIF + EIF). (DOCX)

S3 Table Associations between CD68+ and CD163+ TAMs with clinicopathologic characteristics in TC (STC + ETC). (DOCX)

S4 Table Associations between CD68+ and CD163+ TAMs with clinicopathologic characteristics in S (SIF + STC). (DOCX)

S5 Table Associations between CD68+ and CD163+ TAMs with clinicopathologic characteristics in E (EIF + ETC). (DOCX)

S6 Table Prognostic role of TAMs on various types of tumors. (DOCX)
