# Supplementary figures and images for: Prognostic Implication of M2 Macrophages Are Determined by the Proportional Balance of Tumor Associated Macrophages and Tumor Infiltrating Lymphocytes in Microsatellite-Unstable Gastric Carcinoma
Source: PLoS One. 2015 Dec 29;10(12):e0144192. doi: 10.1371/journal.pone.0144192 (PMC4699826; doi:10.1371/journal.pone.0144192)

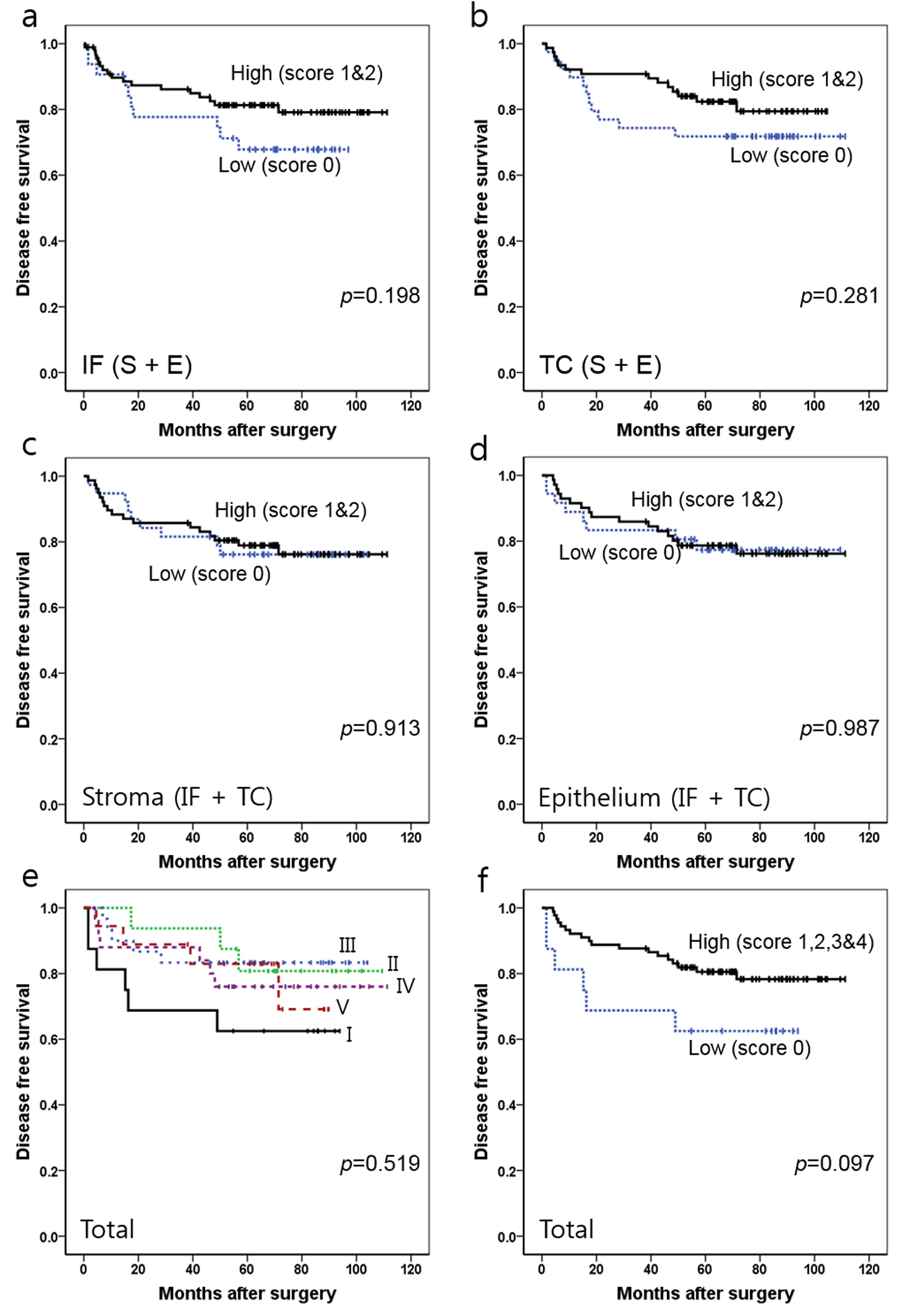

Supplement: S1 Fig — (a) Survival curves of the low density (score 0) (n = 32) vs. high density (score 1 and 2) (n = 87) groups in IF (SIF + EIF). (b) Survival curves of the low density (score 0) (n = 26) vs. high density (score 1 and 2) (n = 82) groups in TC (STC + ETC). (c) Survival curves of the low density (score 0) (n = 38) vs. high density (score 1 and 2) (n = 74) groups in S (SIF+STC). (d) Survival curves of the low density (score 0) (n = 36) vs. high density (score 1 and 2) (n = 68) groups in E (EIF+ETC). (e) Survival curves of five subgroups determined by the total score in four combined areas (SIF + STC + EIF + ETC) (I, score 0 (n = 16); II, score 1 (n = 16); III, score 2 (n = 29); IV, score 3 (n = 24) and V, score 4 (n = 17)). (f) Survival curves of the low density (score 0) (n = 16) vs. high density (score 1–4) (n = 86) groups in four combined areas (SIF + STC + EIF + ETC). Abbreviations: TC, tumor center; IF, invasive front; S, stroma; E, epithelium. (TIF) [file pone.0144192.s001.tif]

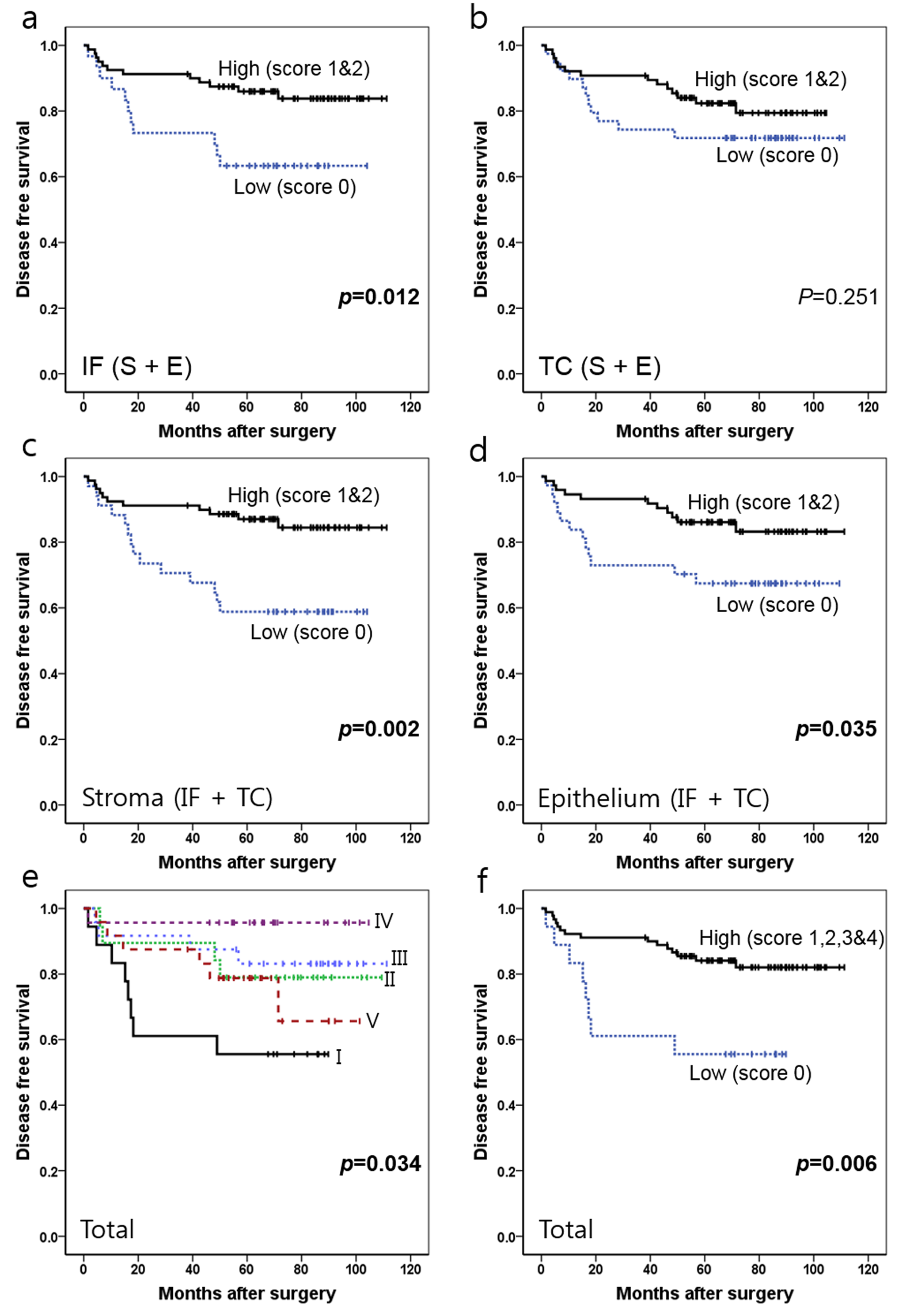

Supplement: S2 Fig — (a) Survival curves of the low density (score 0) (n = 30) vs. high density (score 1 and 2) (n = 77) groups in IF (SIF + EIF). (b) Survival curves of the low density (score 0) (n = 39) vs. high density (score 1 and 2) (n = 73) groups in TC (STC + ETC). (c) Survival curves of the low density (score 0) (n = 34) vs. high density (score 1 and 2) (n = 76) groups in S (SIF+STC). (d) Survival curves of the low density (score 0) (n = 37) vs. high density (score 1 and 2) (n = 70) groups in E (EIF+ETC). (e) Survival curves of five subgroups determined by the total score in four combined areas (SIF + STC + EIF + ETC) (I, score 0 (n = 18); II, score 1 (n = 19); III, score 2 (n = 24); IV, score 3 (n = 22) and V, score 4 (n = 22)). (f) Survival curves of the low density (score 0) (n = 18) vs. high density (score 1–4) (n = 87) groups in four combined areas (SIF + STC + EIF + ETC). Abbreviations: TC, tumor center; IF, invasive front; S, stroma; E, epithelium. (TIF) [file pone.0144192.s002.tif]
