# Supplementary material for: Prognostic Implication of M2 Macrophages Are Determined by the Proportional Balance of Tumor Associated Macrophages and Tumor Infiltrating Lymphocytes in Microsatellite-Unstable Gastric Carcinoma
Source: PLoS One. 2015 Dec 29;10(12):e0144192. doi: 10.1371/journal.pone.0144192 (PMC4699826; doi:10.1371/journal.pone.0144192)
Supplement: S1 Table — (DOCX) [file pone.0144192.s003.docx]

**S1 Table.** Correlation of CD68+ and CD163+ TAMs density according to each compartment in each region in MSI-H GCs.

|  |  | CD68  Stroma in IF | CD68  Epithelium in IF | CD68  Stroma in TC | CD68  Epithelium in TC | CD163  Stroma in IF | CD163  Epithelium in IF | | CD163  Stroma in TC | CD163  Epithelium in TC |
| --- | --- | --- | --- | --- | --- | --- | --- | --- | --- | --- |
| CD68  Stroma in IF | r | (-) | 0.283 | 0.470 | 0.156 | 0.760 | 0.175 | 0.182 | | 0.033 |
|  | p value |  | 0.001 | <0.001 | 0.073 | <0.001 | 0.044 | 0.033 | | 0.701 |
| CD68  Epithelium in IF | r |  | (-) | 0.255 | 0.719 | 0.212 | 0.715 | 0.111 | | 0.506 |
|  | p value |  |  | 0.003 | <0.001 | 0.013 | <0.001 | 0.200 | | <0.001 |
| CD68  Stroma in TC | r |  |  | (-) | 0.306 | 0.502 | 0.358 | 0.515 | | 0.327 |
|  | p value |  |  |  | <0.001 | <0.001 | <0.001 | <0.001 | | <0.001 |
| CD68  Epithelium in TC | r |  |  |  | (-) | 0.098 | 0.597 | 0.170 | | 0.650 |
|  | p value |  |  |  |  | 0.264 | <0.001 | 0.049 | | <0.001 |
| CD163  Stroma in IF | r |  |  |  |  | (-) | 0.300 | 0.352 | | 0.185 |
|  | p value |  |  |  |  |  | <0.001 | <0.001 | | 0.030 |
| CD163  Epithelium in IF | r |  |  |  |  |  | (-) | 0.496 | | 0.770 |
|  | p value |  |  |  |  |  |  | <0.001 | | <0.001 |
| CD163  Stroma in TC | r |  |  |  |  |  |  | (-) | | 0.592 |
|  | p value |  |  |  |  |  |  |  | | <0.001 |
| CD163  Epithelium in TC | r |  |  |  |  |  |  |  | | (-) |
|  | p value |  |  |  |  |  |  |  | |  |

r, correlation coefficient *Abbreviations* : TAM, tumor associated macrophages; IF, invasive front; TC, tumor center
