# Supplementary material for: Prognostic Implication of M2 Macrophages Are Determined by the Proportional Balance of Tumor Associated Macrophages and Tumor Infiltrating Lymphocytes in Microsatellite-Unstable Gastric Carcinoma
Source: PLoS One. 2015 Dec 29;10(12):e0144192. doi: 10.1371/journal.pone.0144192 (PMC4699826; doi:10.1371/journal.pone.0144192)
Supplement: S2 Table — (DOCX) [file pone.0144192.s004.docx]

**S2 Table.** Associations between CD68+ and CD163+ TAMs with clinicopathologic characteristics in IF (SIF + EIF).

| Parameters | Case no. | CD68+ TAMs^a^ | | | Case no. | CD163+ TAMs^a^ | | |
| --- | --- | --- | --- | --- | --- | --- | --- | --- |
|  |  | Low | High | *P* value |  | Low | High | *P* value |
| Gender  Male  Female | 74  60 | 26 (59.1%)  18 (40.9%) | 48 (53.3%)  42 (46.7%) | 0.582 | 73  59 | 21 (53.8%)  18 (46.2%) | 52 (55.9%)  41 (44.1%) | 0.850 |
| Age (years)  ≤60  >60 | 40  94 | 16 (36.4%)  28 (63.6%) | 24 (26.7%)  66 (73.3%) | 0.315 | 38  94 | 11 (28.2%)  28 (71.8%) | 27 (29.0%)  66 (71.0%) | 1.000 |
| Body mass index (BMI)^b^  Low  High | 75  65 | 25 (56.8%)  19 (43.2%) | 50 (52.1%)  46 (47.9%) | 0.715 | 67  61 | 25 (67.6%)  12 (32.4%) | 42 (46.2%)  49 (53.8%) | 0.033 |
| Site  Upper  Middle  Lower | 9  22  103 | 2 (4.5%)  7 (15.9%)  35 (79.5%) | 7 (7.8%)  15 (16.7%)  68 (75.6%) | 0.767 | 9  22  101 | 1 (2.6%)  7 (17.9%)  31 (79.5%) | 8 (8.6%)  15 (16.1%)  70 (75.3%) | 0.452 |
| AJCC Stage  I/II  III | 89  45 | 32 (72.7%)  12 (27.3%) | 57 (63.3%)  33 (36.7%) | 0.333 | 88  44 | 24 (61.5%)  15 (38.5%) | 64 (68.8%)  29 (31.2%) | 0.426 |
| Tumor depth  T2  T3/T4 | 37  97 | 16 (36.4%)  28 (63.6%) | 21 (23.3%)  69 (76.7%) | 0.150 | 37  95 | 12 (30.8%)  27 (69.2%) | 25 (26.9%)  68 (73.1%) | 0.675 |
| LN metastasis^b^  Absent  Present | 86  47 | 31 (70.5%)  13 (29.5%) | 55 (61.8%)  34 (38.2%) | 0.344 | 85  46 | 24 (61.5%)  15 (38.5%) | 61 (66.3%)  31 (33.7%) | 0.690 |
| WHO classification  WD/MD  PD | 70  64 | 31 (70.5%)  13 (29.5%) | 39 (43.3%)  51 (56.7%) | 0.003 | 69  63 | 26 (66.7%)  13 (33.3%) | 43 (46.2%)  50 (53.8%) | 0.037 |
| Lymphatic invasion  Absent  Present | 50  84 | 14 (31.8 %)  30 (68.2%) | 36 (40.0%)  54 (60.0%) | 0.447 | 49  83 | 11 (28.2%)  28 (71.8%) | 38 (40.9%)  55 (59.1%) | 0.236 |
| Vascular invasion  Absent  Present | 114  20 | 36 (81.8%)  8 (18.2%) | 78 (86.7%)  12 (13.3%) | 0.452 | 112  20 | 32 (82.1%)  7 (17.9%) | 80 (86.0%)  13 (14.0%) | 0.599 |
| Perineural invasion  Absent  Present | 88  46 | 30 (68.2%)  14 (31.8%) | 58 (64.4%)  32 (35.6%) | 0.703 | 98  44 | 24 (61.5%)  15 (38.5%) | 64 (68.8%)  29 (31.2%) | 0.426 |
| Lauren classification  Intestinal  Diffuse | 76  58 | 32 (72.7%)  12 (27.3%) | 44 (48.9%)  46 (51.5%) | 0.010 | 75  57 | 25 (64.1%)  14 (35.9%) | 50 (53.8%)  43 (46.2%) | 0.337 |
| Ming’s classification  Expanding  Infiltrative | 36  98 | 13 (29.5%)  31 (70.5%) | 23 (25.6%)  67 (74.4%) | 0.680 | 36  96 | 7 (17.9%)  32 (82.1%) | 29 (31.2%)  64 (68.8%) | 0.138 |
| *MLH1* expression^c^  Retained  Loss | 14  112 | 3 (7.5%)  37 (92.5%) | 12 (12.8%)  75 (87.2%) | 0.546 | 12  112 | 3 (8.1%)  34 (91.9%) | 9 (10.3%)  78 (89.7%) | 1.000 |
| *MSH2* expression^c^  Retained  Loss | 117  9 | 38 (95.0%)  2 (5.0%) | 79 (91.9%)  7 (8.1%) | 0.720 | 115  9 | 35 (94.6%)  2 (5.4%) | 80 (92.0%)  7 (8.0%) | 0.723 |

^a^Included only for patients with data available on TMA.

^b^Information only for patients with available clinicopathlogic data.

^c^Included only for patients with data available of immunohistochemistry.

*Abbreviations* : TAM, tumor associated macrophage; IF, invasive front; SIF, Stromal TAMs density in invasive front; EIF, Epithelial TAMs density in invasive front; LN, lymph node
