# Supplementary material for: Prognostic Implication of M2 Macrophages Are Determined by the Proportional Balance of Tumor Associated Macrophages and Tumor Infiltrating Lymphocytes in Microsatellite-Unstable Gastric Carcinoma
Source: PLoS One. 2015 Dec 29;10(12):e0144192. doi: 10.1371/journal.pone.0144192 (PMC4699826; doi:10.1371/journal.pone.0144192)
Supplement: S3 Table — (DOCX) [file pone.0144192.s005.docx]

**S3 Table.** Associations between CD68+ and CD163+ TAMs with clinicopathologic characteristics in TC (STC + ETC).

| Parameters | Case no. | CD68+ TAMs^a^ | | | Case no. | CD163+ TAMs^a^ | | |
| --- | --- | --- | --- | --- | --- | --- | --- | --- |
|  |  | Low | High | *P* value |  | Low | High | *P* value |
| Gender  Male  Female | 72  61 | 21 (56.8%)  16 (43.2%) | 51 (53.1%)  45 (46.9%) | 0.846 | 74  64 | 35 (67.3%)  17 (32.7%) | 39 (45.3%)  47 (54.7%) | 0.014 |
| Age (years)  ≤60  >60 | 36  97 | 8 (21.6%)  29 (78.4%) | 28 (29.2%)  68 (70.8%) | 0.514 | 40  98 | 15 (28.8%)  37 (71.2%) | 25 (29.1%)  61 (70.9%) | 1.000 |
| Body mass index (BMI)^b^  Low  High | 67  63 | 18 (48.6%)  19 (51.4%) | 49 (52.7%)  44 (47.3%) | 0.702 | 69  65 | 26 (51.0%)  25 (49.0%) | 43 (51.8%)  40 (48.2%) | 1.000 |
| Site  Upper  Middle  Lower | 11  22  100 | 1 (2.7%)  7 (18.9%)  29 (78.4%) | 10 (10.4%)  15 (15.6%)  71 (74.0%) | 0.339 | 11  22  105 | 2 (3.8%)  8 (15.4%)  42 (80.8%) | 9 (10.5%)  14 (16.3%)  63 (73.3%) | 0.361 |
| AJCC Stage  I/II  III | 86  47 | 25 (67.6%)  12 (32.4%) | 61 (63.5%)  35 (36.5%) | 0.692 | 90  48 | 35 (67.3%)  17 (32.7%) | 55 (64.0%)  31 (36.0%) | 0.716 |
| Tumor depth  T2  T3/T4 | 36  97 | 12 (32.4%)  25 (67.6%) | 24 (25.0%)  72 (75.0%) | 0.392 | 38  100 | 16 (30.8%)  36 (69.2%) | 22 (25.6%)  64 (74.4%) | 0.558 |
| LN metastasis^b^  Absent  Present | 85  47 | 25 (67.6%)  12 (32.4%) | 60 (63.2%)  35 (36.8%) | 0.690 | 88  49 | 36 (69.2%)  16 (30.8%) | 52 (61.2%)  33 (38.8%) | 0.364 |
| WHO classification  WD/MD  PD | 66  67 | 22 (59.5%)  15 (40.5%) | 44 (45.8%)  52 (54.2%) | 0.179 | 70  68 | 33 (63.5%)  19 (36.5%) | 37 (43.0%)  49 (57.0%) | 0.023 |
| Lymphatic invasion  Absent  Present | 52  81 | 9 (24.3 %)  28 (75.7%) | 43 (44.8%)  53 (55.2%) | 0.046 | 52  86 | 17 (32.7%)  35 (67.3%) | 35 (40.7%)  51 (59.3%) | 0.371 |
| Vascular invasion  Absent  Present | 114  19 | 28 (75.7%)  9 (24.3%) | 86 (89.6%)  10 (10.4%) | 0.053 | 118  20 | 42 (80.8%)  10 (19.2%) | 76 (88.4%)  10 (11.6%) | 0.225 |
| Perineural invasion  Absent  Present | 86  47 | 22 (59.5%)  15 (40.5%) | 64 (66.7%)  32 (33.3%) | 0.554 | 91  47 | 31 (59.6%)  21 (40.4%) | 60 (69.8%)  26 (30.2%) | 0.267 |
| Lauren classification  Intestinal  Diffuse | 74  59 | 27 (73.0%)  10 (27.0%) | 47 (49.0%)  49 (51.0%) | 0.019 | 77  61 | 38 (73.1%)  14 (26.9%) | 39 (45.3%)  47 (54.7%) | 0.002 |
| Ming’s classification  Expanding  Infiltrative | 36  97 | 9 (24.3%)  28 (75.7%) | 27 (28.1%)  69 (71.9%) | 0.828 | 36  102 | 15 (28.8%)  37 (71.2%) | 21 (24.4%)  65 (75.6%) | 0.689 |
| *MLH1* expression^c^  Retained  Loss | 14  112 | 4 (11.4%)  31 (88.6%) | 10 (11.0%)  81 (89.0%) | 1.000 | 14  118 | 4 (7.8%)  47 (92.2%) | 10 (12.3%)  71 (87.7%) | 0.565 |
| *MSH2* expression^c^  Retained  Loss | 117  9 | 34 (97.1%)  1 (2.9%) | 83 (91.0%)  8 (8.8%) | 0.443 | 123  9 | 50 (98.0%)  1 (2.0%) | 73 (90.1%)  8 (9.9%) | 0.152 |

^a^Included only for patients with data available on TMA.

^b^Information only for patients with available clinicopathlogic data.

^c^Included only for patients with data available of immunohistochemistry.

*Abbreviations* : TAM, tumor associated macrophage; TC, tumor center; STC, Stromal TAMs density in tumor center; ETC, Epithelial TAMs density in tumor center; LN, lymph node
