# Supplementary material for: Prognostic Implication of M2 Macrophages Are Determined by the Proportional Balance of Tumor Associated Macrophages and Tumor Infiltrating Lymphocytes in Microsatellite-Unstable Gastric Carcinoma
Source: PLoS One. 2015 Dec 29;10(12):e0144192. doi: 10.1371/journal.pone.0144192 (PMC4699826; doi:10.1371/journal.pone.0144192)
Supplement: S4 Table — (DOCX) [file pone.0144192.s006.docx]

**S4 Table.** Associations between CD68+ and CD163+ TAMs with clinicopathologic characteristics in S (SIF + STC).

| Parameters | Case no. | CD68+ TAMs^a^ | | | Case no. | CD163+ TAMs^a^ | | |
| --- | --- | --- | --- | --- | --- | --- | --- | --- |
|  |  | Low | High | *P* value |  | Low | High | *P* value |
| Gender  Male  Female | 76  63 | 27 (55.5%)  22 (44.9%) | 49 (54.4%)  41 (45.6%) | 1.000 | 73  63 | 25 (58.7%)  18 (41.9%) | 48 (51.6%)  45 (48.4%) | 0.580 |
| Age (years)  ≤60  >60 | 41  98 | 17 (34.7%)  32 (65.3%) | 24 (26.7%)  66 (73.3%) | 0.337 | 41  95 | 12 (27.9%)  31 (72.1%) | 29 (31.2%)  64 (68.8%) | 0.841 |
| Body mass index (BMI)^b^  Low  High | 69  67 | 26 (54.2%)  22 (45.8%) | 43 (48.9%)  45 (51.1%) | 0.597 | 67  65 | 28 (66.7&)  14 (33.3%) | 39 (43.3%)  51 (56.7%) | 0.015 |
| Site  Upper  Middle  Lower | 11  22  106 | 3 (6.1%)  6 (12.2%)  40 (81.6%) | 8 (8.9%)  16 (17.8%)  69 (73.3%) | 0.547 | 10  22  104 | 1 (2.3%)  5 (11.6%)  37 (86.0%) | 9 (9.7%)  17 (18.3%)  67 (72.0%) | 0.156 |
| AJCC Stage  I/II  III | 90  49 | 33 (67.3%)  16 (32.7%) | 57 (63.3%)  33 (36.7%) | 0.712 | 88  48 | 26 (60.5%)  17 (39.5%) | 62 (66.7%)  31 (33.3%) | 0.563 |
| Tumor depth  T2  T3/T4 | 39  100 | 16 (32.7%)  33 (67.3%) | 23 (25.6%)  67 (74.4%) | 0.431 | 37  99 | 14 (32.6%)  29 (67.4%) | 23 (24.7%)  70 (75.3%) | 0.408 |
| LN metastasis^b^  Absent  Present | 87  51 | 33 (67.3%)  16 (32.7%) | 54 (60.7%)  35 (39.3%) | 0.467 | 86  49 | 26 (60.5%)  17 (39.5%) | 60 (65.2%)  32 (34.8%) | 0.701 |
| WHO classification  WD/MD  PD | 73  66 | 24 (49.0%)  25 (51.0%) | 49 (54.4%)  41 (45.6%) | 0.596 | 69  67 | 22 (51.2%)  21 (48.8%) | 47 (50.5%)  46 (49.5%) | 1.000 |
| Lymphatic invasion  Absent  Present | 52  87 | 17 (34.7 %)  32 (65.3%) | 35 (38.9%)  55 (61.1%) | 0.715 | 50  86 | 12 (27.9%)  31 (72.1%) | 38 (40.9%)  55 (59.1%) | 0.182 |
| Vascular invasion  Absent  Present | 118  21 | 42 (85.7%)  7 (14.3%) | 76 (84.4%)  14 (15.6%) | 1.000 | 116  20 | 35 (81.4%)  8 (18.6%) | 81 (87.1%)  12 (12.9%) | 0.438 |
| Perineural invasion  Absent  Present | 90  49 | 28 (57.1%)  21 (42.9%) | 62 (68.9%)  28 (31.1%) | 0.195 | 88  48 | 23 (53.5%)  20 (46.5%) | 65 (69.9%)  28 (30.1%) | 0.082 |
| Lauren classification  Intestinal  Diffuse | 78  61 | 28 (57.1%)  21 (42.9%) | 50 (55.6%)  40 (44.4%) | 1.000 | 76  60 | 25 (58.1%)  18 (41.9%) | 51 (54.8%)  42 (45.2%) | 0.853 |
| Ming’s classification  Expanding  Infiltrative | 37  102 | 12 (24.5%)  37 (75.5%) | 25 (27.8%)  65 (72.2%) | 0.841 | 34  102 | 9 (20.9%)  34 (79.1%) | 25 (26.9%)  68 (73.1%) | 0.527 |
| *MLH1* expression^c^  Retained  Loss | 16  116 | 6 (13.0%)  40 (87.0%) | 10 (11.6%)  76 (88.4%) | 0.787 | 15  115 | 4 (9.8%)  37 (90.2%) | 11 (12.4%)  78 (87.6%) | 0.775 |
| *MSH2* expression^c^  Retained  Loss | 123  9 | 44 (95.7%)  2 (4.9%) | 79 (91.9%)  7 (8.1%) | 0.334 | 121  9 | 40 (97.6%)  1 (2.4%) | 81 (91.0%)  8 (9.0%) | 0.271 |

^a^Included only for patients with data available on TMA.

^b^Information only for patients with available clinicopathlogic data.

^c^Included only for patients with data available of immunohistochemistry.

*Abbreviations* : TAM, tumor associated macrophage; S, stroma; SIF, Stromal TAMs density in invasive front; STC, Stromal TAMs density in tumor center; LN, lymph node
