# Supplementary material for: Prognostic Implication of M2 Macrophages Are Determined by the Proportional Balance of Tumor Associated Macrophages and Tumor Infiltrating Lymphocytes in Microsatellite-Unstable Gastric Carcinoma
Source: PLoS One. 2015 Dec 29;10(12):e0144192. doi: 10.1371/journal.pone.0144192 (PMC4699826; doi:10.1371/journal.pone.0144192)
Supplement: S5 Table — (DOCX) [file pone.0144192.s007.docx]

**S5 Table.** Associations between CD68+ and CD163+ TAMs with clinicopathologic characteristics in E (EIF + SIF).

| Parameters | Case no. | CD68+ TAMs^a^ | | | Case no. | CD163+ TAMs^a^ | | |
| --- | --- | --- | --- | --- | --- | --- | --- | --- |
|  |  | Low | High | *P* value |  | Low | High | *P* value |
| Gender  Male  Female | 70  58 | 30 (57.7%)  22 (42.3%) | 40 (52.6%)  36 (47.4%) | 0.593 | 73  60 | 34 (64.2%)  19 (35.8%) | 38 (48.1%)  41 (51.9%) | 0.077 |
| Age (years)  ≤60  >60 | 35  93 | 13 (25.0%)  39 (75.0%) | 22 (28.9%)  54 (71.1%) | 0.689 | 38  94 | 16 (30.2%)  37 (69.8%) | 22 (27.8%)  57 (72.2%) | 0.845 |
| Body mass index (BMI)^b^  Low  High | 65  59 | 24 (46.2%)  28 (53.8%) | 41 (56.9%)  31 (43.1%) | 0.276 | 67  61 | 29 (55.8%)  23 (44.2%) | 38 (50.0%)  38 (50.0%) | 0.590 |
| Site  Upper  Middle  Lower | 9  22  97 | 2 (3.8%)  13 (25.0%)  37 (71.2%) | 7 (9.2%)  9 (11.8%)  60 (78.9%) | 0.099 | 9  22  101 | 2 (3.8%)  11 (20.8%)  40 (75.5%) | 7 (8.9%)  11 (13.9%)  61 (77.2%) | 0.349 |
| AJCC Stage  I/II  III | 85  43 | 36 (69.2%)  16 (30.8%) | 49 (64.5%)  27 (35.5%) | 0.704 | 88  44 | 34 (64.2%)  19 (35.8%) | 54 (68.4%)  25 (31.6%) | 0.707 |
| Tumor depth  T2  T3/T4 | 35  93 | 17 (32.7%)  35 (67.3%) | 18 (23.7%)  58 (76.3%) | 0.314 | 37  95 | 19 (35.8%)  34 (64.2%) | 18 (22.8%)  61 (77.2%) | 0.116 |
| LN metastasis^b^  Absent  Present | 83  44 | 36 (69.2%)  16 (30.8%) | 47 (62.7%)  28 (37.3%) | 0.570 | 85  46 | 32 (60.4%)  21 (39.6%) | 53 (67.9%)  25 (32.1%) | 0.456 |
| WHO classification  WD/MD  PD | 63  65 | 36 (69.2%)  16 (30.8%) | 27 (35.5%)  49 (64.5%) | <0.001 | 68  64 | 37 (69.8%)  16 (30.2%) | 31 (39.2%)  48 (60.8%) | 0.001 |
| Lymphatic invasion  Absent  Present | 50  78 | 20 (38.5 %)  32 (61.5%) | 30 (39.5%)  46 (60.5%) | 1.000 | 49  83 | 14 (26.4%)  39 (73.6%) | 35 (44.3%)  44 (55.7%) | 0.044 |
| Vascular invasion  Absent  Present | 109  19 | 40 (76.9%)  12 (23.1%) | 69 (90.8%)  7 (9.2%) | 0.042 | 112  20 | 42 (79.2%)  11 (20.8%) | 70 (88.6%)  9 (11.4%) | 0.215 |
| Perineural invasion  Absent  Present | 84  44 | 32 (61.5%)  20 (38.5%) | 52 (68.4%)  24 (31.6%) | 0.453 | 89  44 | 38 (64.8%)  19 (35.2%) | 54 (68.4%)  25 (31.6%) | 0.707 |
| Lauren classification  Intestinal  Diffuse | 71  57 | 42 (80.8%)  10 (19.2%) | 29 (38.2%)  47 (61.8%) | <,<0.001 | 75  57 | 41 (77.4%)  12 (22.6%) | 34 (43.0%)  45 (57.0%) | <0.001 |
| Ming’s classification  Expanding  Infiltrative | 35  93 | 13 (25.0%)  39 (75.0%) | 22 (28.9%)  54 (71.1%) | 0.689 | 36  96 | 15 (28.3%)  38 (71.7%) | 21 (26.6%)  58 (73.4%) | 0.844 |
| *MLH1* expression^c^  Retained  Loss | 13  108 | 4 (8.2%)  45 (91.8%) | 9 (12.5%)  63 (87.5%) | 0.557 | 12  113 | 5 (9.6%)  47 (90.4%) | 7 (9.6%)  66 (90.4%) | 1.000 |
| *MSH2* expression^c^  Retained  Loss | 112  9 | 47 (95.9%)  2 (4.1%) | 65 (90.3%)  7 (9.7%) | 0.481 | 116  9 | 49 (94.2%)  3 (5.8%) | 67 (91.8%)  6 (8.2%) | 0.734 |

^a^Included only for patients with data available on TMA.

^b^Information only for patients with available clinicopathlogic data.

^c^Included only for patients with data available of immunohistochemistry.

*Abbreviations* : TAM, tumor associated macrophage; E, epithelium; EIF, Epithelial TAMs density in invasive front; ETC, Epithelial TAMs density in tumor center; LN, lymph node
