# Supplementary material for: Prognostic Implication of M2 Macrophages Are Determined by the Proportional Balance of Tumor Associated Macrophages and Tumor Infiltrating Lymphocytes in Microsatellite-Unstable Gastric Carcinoma
Source: PLoS One. 2015 Dec 29;10(12):e0144192. doi: 10.1371/journal.pone.0144192 (PMC4699826; doi:10.1371/journal.pone.0144192)
Supplement: S6 Table — (DOCX) [file pone.0144192.s008.docx]

**S6 Table.** Prognostic role of TAMs on various types of tumors.

| **Author** | **Tumor type** | **Analyzed molecular marker** | **Methods** | **Conclusion** |
| --- | --- | --- | --- | --- |
| Reinartz S et al | Ovarian cancer | CD14, CCR7, CD206, CD64, CD16, CD32 and HLA-DR | Fluorescence activated cell sorting (FACS), cell culture | Surface expression of the M2 marker CD163 on TAMs was inversely associated with RFS. CD163 expression correlated with the ascites levels of IL-6 and IL-10, and their ascites levels was inversely associated with RFS. |
| Bao-xiang Pei et al. | Non-small cell lung cancer | CD68, CSF-1, IL-6 | IHC | Co-expression of CD68, CSF-1 and IL-6 was significant negative prognostic factor of survival.  The 5-year survival rate in the CD68-negative and CSF-1 and IL-6 positive group was better than the group with CD68, CSF-1 and IL-6 positive group. |
| Kubler K et al. | Endometrial adenocarcinoma | CD163, FoxP3, D2-40 | IHC | Increased CD163+ TAMs was proportionally associated with advanced FIGO stage, high tumor grade, increased lymph vessel density, lymphovascular invasion and lymph node metastasis. |
| Zhang Y et al. | Breast cancer | CD68 | IHC | Breast cancer patients with a high density of TAMs had significantly lower rates of disease-free survival and 5-year overall survival than patients with low density of TAMs.  High-infiltration of TAMs indicated worse survival rate for node-negative breast cancer. |
| Yuan ZY et al. | Breast cancer | CD68 | IHC | High infiltrating TAMs are a significant unfavorable prognostic factor for patients with triple negative breast cancer. |
| Tham M et al. | Melanoma | CD68, CD271, CD45, CD3, CD4, CD8, CD19, CD11b, NK1.1, CD34, CD115 | Cell culture, Mice xenotransplantation (immune competent RETAAD mice mode) | TAMs interact with the tumor initiating cells (TIC) population from RETAAD tumors.  Macrophage-derived TGFβ and polyamines are essential for TIC survival and resistance of chemotherapeutic drugs. |
| Kumagai S et al. | Lung adenocarcinoma | CD68, peripheral monocyte count | IHC | High monocyte count predict poor RFS and OS.  CD68+ TAMs in primary tumors correlated with peripheral monocyte counts. |
| Yang L et al. | Lung cancer (malignant pleural effusion) | CD163, CD14, Arginase-1, IL-10,TGF-β, IL-10, TNF-α, iNOS, CCL2, CCL21, CXCL12 | Flow cytometry, real-time PCR, PA-MSHA treatment in vitro, NK cell cytotoxicity assay, Anti-TLR blocking antibody array | Significant accumulation of CD163+ TAMs in MPE is closely related with poor prognosis. PA-MSHA re-educated CD163+ TAMs to M1 macrophages in MPE via TLR-mediated pathway. |
| Behnes CL et al. | Papillary renal cell carcinoma | CD68, CD163, M-CSF, Ki-67, CD31 | IHC | A dense infiltrate of CD163+ M2-type TAM and high M-CSF expression in tumor cells are major features of type II papillary RCC which shows worse prognosis than type I papillary RCC. |
| Dannenmann SR et al. | Clear cell renal cell carcinoma | CD68, FoxP3, CD163, IRF4, FN1, CD11b | Flow cytometry, FACs, polyclonal stimulation and intracellular cytokine stain (ICS), qRT-PCR | High level of infiltration of Tregs and TAMs in ccRCC correlates with reduced survival.  M2 TAMs induce the skewing of tumor-infiltrating T cells toward a more regulated phenotype. |
| Wang et al. | Gastric cancer | CD68, FoxP3 | IHC | Intra-tumoral infiltrating CD68+ TAMs are independent good prognostic factors. |
| Wu et al. | Gastric cancer | CD68 | IHC | CD68+ TAMs promote angiogenesis and lymphagiogenesis of GCs. |
| Ishigami et al. | Gastric cancer | CD68 | IHC | Patients with high count of CD68+ TAMs had poorer surgical outcomes. |
| Zhang et al. | Gastric cancer | CD68, CD11c, C206 | IHC | CD68+ TAMs were found to have no prognostic impact on OS.  High density of CD11c+ M1 polarized TAMs and low density of CD206+ M2 polarized TAMs indicated better OS. |
| Edin et al. | Colorectal carcinoma (CRC) | iNOs, CD163 | IHC | An increased infiltration of macrophages with a M1 phenotype at the tumor front is accompanied by a concomitant increase of M2 phenotype macrophages in a stage dependent manner and is associated with better prognosis in CRCs. |
| Forssell J et al. | CRCs | CD68 | IHC, in vitro coculture experiments | CD68+ TAMs turned out as an independent favorable prognostic marker in CRCs.  In vitro coculture experiments, high ratio of macrophages to colon cancer cells inhibited cancer cell growth. |
| Algars A et al. | CRCs | CD68, CLEVER-1/Stabilin-1, podoplanin | IHC | A high number of CLEVER-1/Stabilin-1(+) M2 TAMs positively correlated with survival in less advanced disease, but in more advanced disease, inverse prognostic correlation was observed between CLEVER-1/Stabilin-1(+) TAMs.  A low number of suppressive intratumoral and to fewer disease relapse exclusively in the liver. |
| Hernandez C et al. | CRCs | CD68, CD163, CD206, Gastrin, Wnt 1 | IHC, cell culture | The expression of gastric peptide (Gastrin) in CRC correlates with a reduced infiltration of M2-macrophages.  Progastrin secretion of Wnt ligands by M2-macophages and increase their ability to induce apoptosis of colon cancer cells. |
| Siew-Min Ong et al. | CRCs | C3, CD68, CD14, IL-6, IL-8, IFN-γ, VEGF, CCL2, CCL3, CCL4, CCL7, CCL8, CXCL9, CXCL10, CXCL12 | Generation of multi-cellular tumor spheroid (MCTS), Flow cytometry, FACS, Real-time PCR, proliferation assay, multiplex microbead immunoassay and ELISA, transwell assay, mixed lymphocyte reaction assay, IHC, IF | TAMs in CRCs were pro-inflammatory and inhibited the proliferation of tumor cells.  TAMs in CRCs produced chemokines that attract T cells particularly type-1 T cells.  TAMs exerted tumor-suppressive effects with the help of T cells. |
